# A potential correlation between adipokines, skeletal muscle function and bone mineral density in middle-aged and elderly individuals

**DOI:** 10.1186/s12944-023-01879-z

**Published:** 2023-07-31

**Authors:** Wenhao Wang, Xuchao Gu, Ziyi Cao, Xiaojun Wang, Yiming Lei, Xiaoli Xu, Shiwen Wang, Tao Wu, Zhijun Bao

**Affiliations:** 1 Shanghai Key Laboratory of Clinical Geriatric Medicine, Huadong Hospital Affiliated to Fudan University, Shanghai, 200040 China; 2 Department of Traditional Chinese Medicine, Huadong Hospital Affiliated to Fudan University, No 221 West Yan-An Road, Shanghai, 200040 China; 3 Department of Gastroenterology, Huadong Hospital Affiliated to Fudan University, Shanghai, 200040 China; 4 Department of Gerontology, Huadong Hospital Affiliated to Fudan University, No 221 West Yan-An Road, Shanghai, 200040 China; 5 Department of Laboratory Medicine, Huadong Hospital Affiliated to Fudan University, No 221 West Yan-An Road, Shanghai, 200040 China

**Keywords:** Skeletal muscle function, Bone mineral density, Adipokine, Inflammation

## Abstract

**Background:**

Evidence exists of a strong association between inflammation and a decrease in skeletal muscle function and bone mineral density (BMD); however, the specific mechanisms of these associations remain unclear. Adipokines, as key regulators of the inflammatory response, may be implicated in these processes. The objective of this study was to explore the potential correlation between adipokines, skeletal muscle function and BMD in middle-aged and elderly individuals.

**Methods:**

A comparative cross-sectional study was carried out at the Huadong Hospital Affiliated with Fudan University (Shanghai, China). A total of 460 middle-aged and elderly individuals were recruited, and 125 were enrolled in the analysis. Their biochemical indices, body composition, skeletal muscle function and BMD were measured. Bioinformatic analysis was also employed to identify potential adipokine targets linked to skeletal muscle function and BMD. To validate these targets, plasma and peripheral blood mononuclear cells (PBMCs) were harvested from these individuals and subjected to western blotting (WB) and enzyme-linked immunosorbent assay (ELISA).

**Results:**

Individuals in this cross-sectional study were categorized into 2 groups according to their median skeletal muscle mass (SMM) (28.8 kg for males and 20.6 kg for females). Individuals with lower SMM exhibited poorer grip strength (*P* = 0.017), longer 5-Times-Sit-to-Stand Test (FTSST) duration (*P* = 0.029), lower total hip BMD (*P* = 0.043), lower femoral neck BMD (*P* = 0.011) and higher levels of inflammatory markers in comparison with individuals with higher SMM. Bioinformatics analysis identified LEP, ADIPOQ, RBP4, and DPP4 as potential adipokine targets associated with skeletal muscle function and BMD. In vitro experiments demonstrated that individuals with decreased skeletal muscle function and BMD expressed higher levels of these adipokines.

**Conclusions:**

Skeletal muscle function is positively correlated with BMD and negatively correlated with levels of inflammatory markers among middle-aged and elderly individuals. Those with lower skeletal muscle function and BMD tend to have a higher expression of LEP, ADIPOQ, RBP4 and DPP4.

**Supplementary Information:**

The online version contains supplementary material available at 10.1186/s12944-023-01879-z.

## Introduction

Decreased skeletal muscle function is a common issue among elderly individuals [1]. Recent studies suggest that lipids and adipokines in skeletal muscle may be responsible for this condition. Intramyocellular lipids (IMCLs) can accumulate in skeletal muscle during aging, which results in inflammation, impaired mitochondrial oxidative phosphorylation and decreased skeletal muscle function [2]. Adipocytes present in skeletal muscle can also interfere with the normal expression of adipokines, resulting in inflammatory reactions [3], catabolism and damage to skeletal muscle regeneration [4]. Inflammation is a risk factor for skeletal muscle health, and PBMCs are associated with the induction and propagation of inflammation. PBMCs are capable of releasing numerous inflammatory mediators and proinflammatory cytokines [5] by producing adipokines or being activated by adipokines [6, 7] to drive ongoing chronic inflammation, ultimately leading to a decline in skeletal muscle function.

Osteoporosis is an age-related disease characterized by decreased bone strength and mass, which poses a serious threat to both the patient’s lifespan and quality of life. According to an epidemiological study, over 50% of American adults aged 50 and above suffer from decreased BMD, and among them, 1/3 of women and 1/5 of men may experience an osteoporotic fracture at some point during their remaining lifetime [8, 9]. Numerous studies have indicated a significant correlation between BMD and skeletal muscle function [10–12]. Skeletal muscle regulates bone metabolism through the endocrine system and mechanical loading, while bones can affect the development of skeletal muscle from local or humoral factors [13]. When the interaction between these tissues is disrupted, both BMD and skeletal muscle function tend to decline simultaneously. Adipokines, which function as modulators of inflammation, have recently been suggested to have a potential adverse effect on this interaction [14, 15].

Decreased skeletal muscle function and osteoporosis are both proinflammatory conditions, and adipokines may promote the activation of inflammation in these processes. Understanding the impact of adipokines on inflammation, skeletal muscle function and BMD may contribute to the development of new treatment strategies against sarcopenia or osteoporosis. In this study, we hypothesized that higher levels of adipokines and inflammatory markers are related to decreased skeletal muscle function and decreased BMD in middle-aged and elderly individuals. Specifically, the relationship between inflammatory markers, skeletal muscle function and BMD was comprehensively assessed by a cross-sectional study. Three public RNA-seq datasets were analyzed using bioinformatics techniques to identify potential adipokine targets in the connection between skeletal muscle function and BMD. Finally, based on the cross-sectional study, the protein levels of LEP and ADIPOQ in PBMCs were evaluated, while the levels of LEP, ADIPOQ, RBP4, DPP4, IL-8 and TNF-α in plasma were evaluated through ELISA so that differences in the expression of these targets among middle-aged and elderly individuals with varying skeletal muscle function and BMD were validated.

## Materials and methods

**Fig. 1:**
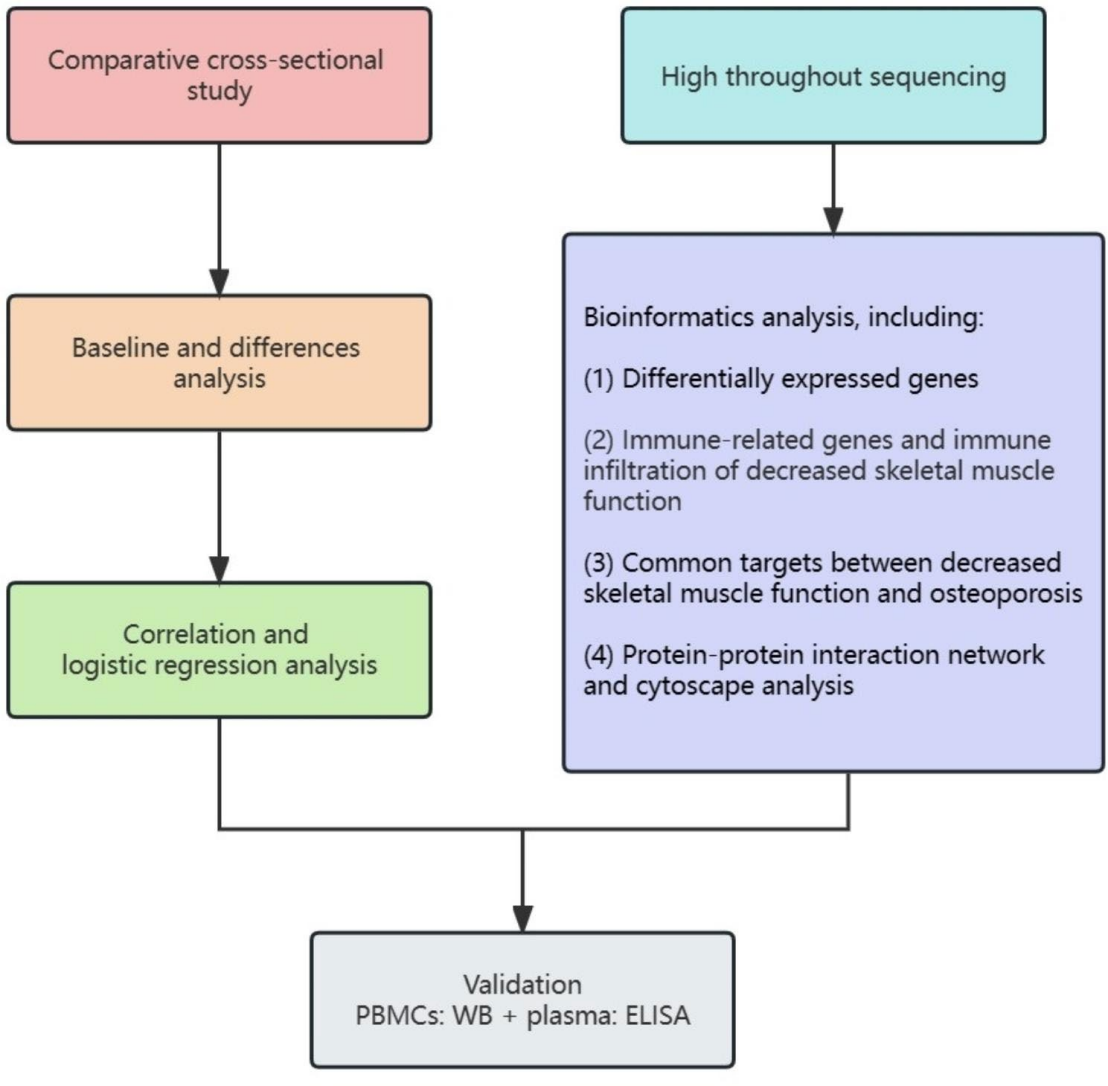
Schematic diagram of the workflow conducted in this study

Our study workflow is shown in Fig. 1.

### Study design of the comparative cross-sectional study

**Fig. 2:**
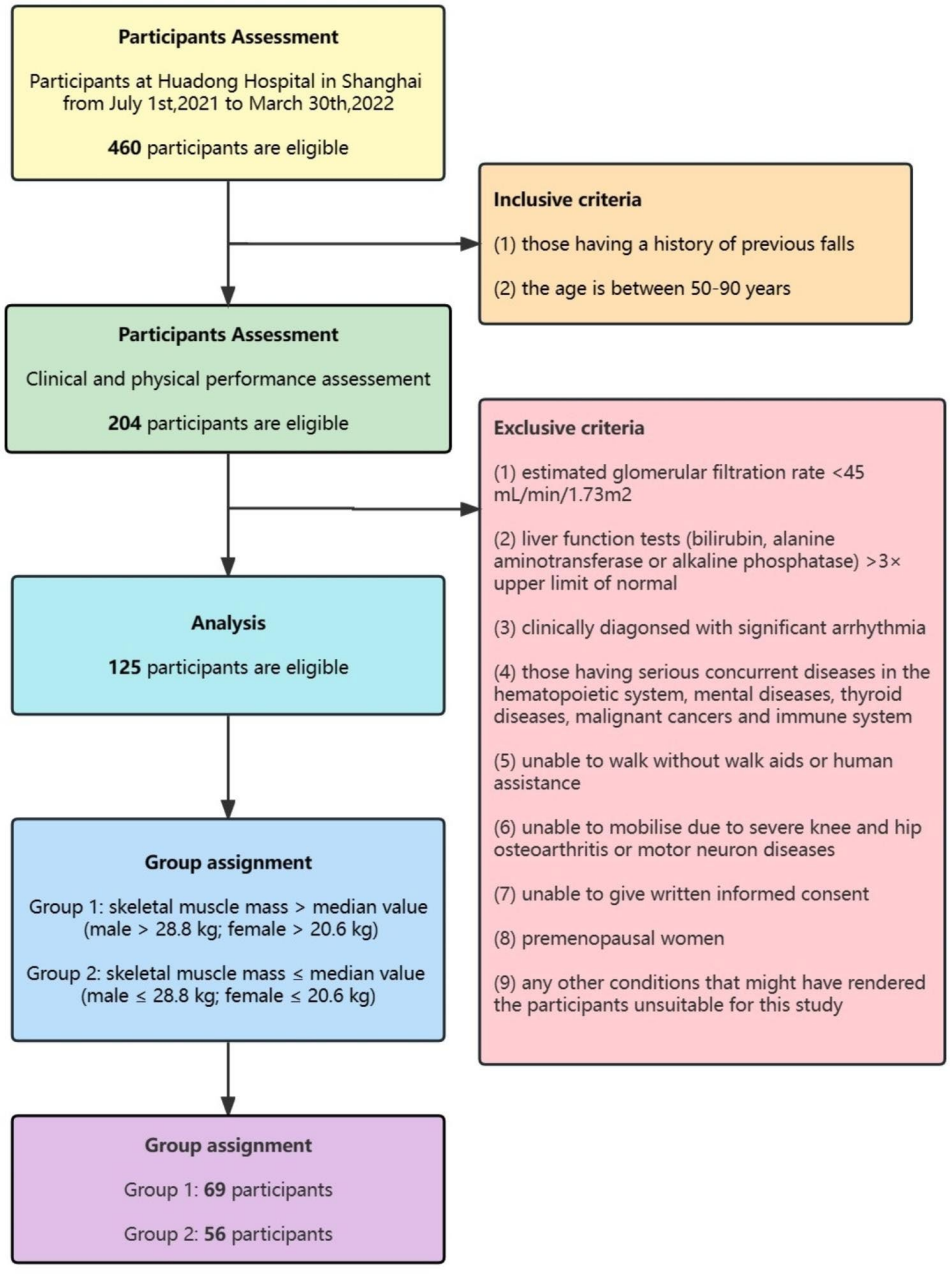
Participant flow in the comparative cross-sectional study

In this study, we conducted a comparative cross-sectional study on middle-aged and elderly individuals (age: 50–90 years) who visited the inpatient department and outpatient department of Huadong Hospital from July 1, 2021, to March 30, 2022. Altogether, 125 eligible individuals were assessed, and the inclusion and exclusion criteria are shown in Fig. 2. Individuals were then grouped according to gender and median SMM. In their respective sexes, those with higher SMM were assigned to group 1, while those with lower SMM were assigned to group 2. Our research protocol received approval from the Ethics Committee of Huadong Hospital (Approval no. 2021K114), and we conducted this work following the Declaration of Helsinki guidelines.

Body composition was assessed using Bioelectrical Impedance Analysis (InBody 770), which included measurements for height, weight, body fat mass, SMM, muscle mass in four extremities, trunk muscle mass, and basal metabolic rate. We calculated appendicular skeletal muscle mass (ASM) by adding up the muscle mass of all four limbs and determined the skeletal muscle index (SMI) by dividing ASM by height squared. We measured skeletal muscle function using grip strength and FTSST. To measure grip strength, we used the CAMRY electronic hand dynamometer and measured the dominant hand’s grip strength in a seated position with the elbow straightened. The FTTST was conducted to evaluate the individuals’ lower limb function. During the test, individuals were instructed to sit at the edge of a chair with their arms folded and repeatedly tap their buttocks against the chair as quickly as possible.

BMD and T scores of the lumbar spine, total hip, and femoral neck were assessed with dual-energy X-ray absorptiometry (DXA). BMD was measured in g/cm^2^. Osteopenia was considered at a T score below − 1, and osteoporosis was considered at a T score <–2.5, according to guidelines released by the World Health Organization.

Blood was sampled from individuals in the morning after a 12-hour fast and analyzed in the laboratory of Huadong Hospital. We measured the estimated glomerular filtration rate (eGFR) to evaluate kidney function using the Chronic Kidney Disease Epidemiology Collaboration equation. Serum creatinine (Scr), blood urea nitrogen (BUN), serum uric acid (SUA), aspartate transaminase (AST), alanine aminotransferase (ALT), alkaline phosphatase (ALP), triglycerides (TG), total cholesterol (TC), high- and low-density lipoprotein (HDL/LDL), glycated hemoglobin (HbA1c), IL-6 and C-reactive protein (CRP) levels were measured. Information about individuals’ history of alcohol consumption and smoking was obtained in face-to-face interviews.

### Data acquisition and preprocessing

Three RNA-seq datasets (GSE111006, GSE111010, and GSE111016) containing skeletal muscle samples of older males were obtained based on the Gene Expression Omnibus (GEO) database. In line with differences in clinical phenotypes, skeletal muscle samples with low skeletal muscle function were assigned to the skeletal muscle function decrease group. In contrast, samples with normal skeletal muscle function were assigned to the control group. Therefore, the skeletal muscle function decrease group consisted of 33 skeletal muscle samples, whereas the control group included 42 skeletal muscle samples.

### Identification of differentially expressed genes (DEGs) and Kyoto Encyclopedia of genes and genomes (KEGG) analysis

Differential expression analysis was carried out with the “limma” R package for identifying DEGs. The cutoff criteria were adj.*P*.Val < 0.05 and |log2FC|>1. The “heatmap” R package was adopted for result visualization. KEGG analysis was conducted with the “clusterProfiler” R package to identify DEG-enriched functions and pathways. The cutoff criteria for this analysis were *P*.Val < 0.05 and |log2FC|>1.

### Immune-related genes (IRGs) and immune cell infiltration analysis

We downloaded 1793 nonredundant IRGs based on the ImmPort database and determined IRGs related to skeletal muscle function using the “ggplot2” R package. In addition, immune cell proportions in muscle biopsy samples were quantified using the CIBERSORT algorithm. The “heatmap” R package was employed to draw a heatmap to visualize the correlation between immune cells and IRGs. The “ggplot2” R package was utilized to compare immune cell infiltration levels between the 2 groups.

### Screening of adipokine targets between skeletal muscle function and BMD

Osteoporosis-related genes were acquired based on the DisGeNET database. Meanwhile, the Venn diagram displayed the shared targets between skeletal muscle function and osteoporosis. We subjected these shared targets to Gene Ontology (GO) annotation with the “clusterProfiler” R package to identify functions and pathways associated with the shared targets in greater detail. We also built a protein–protein interaction (PPI) network for common targets using the STRING database (https://cn.string-db.org/), with a confidence score of > 0.4 as a cutoff criterion. Cytoscape 3.8.0 software was used to further screen adipokine targets and visualize them.

### Blood sampling

Peripheral blood samples were collected from eligible individuals. To obtain plasma, the collected samples were then added to EDTA-coated tubes for a 5-min centrifugation at 500×*g*. PBMCs were extracted in peripheral blood through Ficoll-Hypaque density gradient centrifugation with Lymphoprep (Stemcell Technologies, Shanghai, China). All stock solutions were preserved at − 80 °C. Individuals in this work were voluntary and provided informed consent.

### Western blotting assay

We utilized RIPA lysis buffer (BioLegend, England) to extract total PBMC proteins, separated the proteins through SDS‒PAGE, and later transferred the proteins to PVDF membranes. Membranes were later subjected to 1-h blocking at ambient temperature and overnight incubation with primary antibodies (LEP rabbit pAb and ADIPOQ rabbit pAb) (1:1000, ABclonal Technology, Wuhan, China) at 4 °C. Thereafter, the membranes were rinsed with TBST (1×TBS and 0.5% Tween-20, pH 7.4) three times (10 min each), followed by secondary antibody incubation (HRP-labeled goat anti-rabbit IgG, ABclonal Technology) at a 1:2000 dilution at ambient temperature for 60 min. Chemiluminescence was conducted, and the results were photographed. Each experiment was repeated three times.

### ELISA

The plasma levels of LEP, ADIPOQ, RBP4, DPP4, IL-8 and TNF-α were measured by ELISA following specific protocols (Elabscience Biotechnology, Wuhan, China). Each standard and sample were loaded together with the corresponding antibodies in all wells in a micro-ELISA plate, and then excessive avidin-HRP-conjugated secondary biotinylated antibodies were added. The nonbinding components were removed by rinsing. Substrates and stop solutions were then added, and absorbance (OD) values were detected using a spectrophotometer at 450 nm. Each assay was conducted three times.

### Statistical analyses

Continuous variables were represented by mean ± SD and analyzed by t test or Mann-Whiney U test, while categorical variables were represented by numbers and percentages and analyzed with a chi-square test. We conducted binary logistic regression analysis and Spearman’s correlation analysis to evaluate the relationship of skeletal muscle parameters with BMD. Stata 16.0 was the statistical software used for this analysis. *P* < 0.05 (two-sided) indicated a significant difference.

## Results

### The association between inflammatory markers, skeletal muscle function and BMD among middle-aged and elderly individuals

**A total of** 125 individuals were included in the present work. The median SMM for males and females was 28.8 and 20.6 kg, respectively. Sixty-nine individuals with higher SMM were assigned to Group 1, and 56 with lower SMM were assigned to Group 2. Table 1 displays the basic characteristics. No significant differences between the 2 groups were noted in alcohol consumption, smoking history, or biochemical indices. Although triglyceride levels were significantly different between the 2 groups (*P* = 0.021), these levels were not found to be related to any parameter of skeletal muscle or BMD values (see Supplementary File 1 for details).

**Table 1:** The baseline characteristics of Group 1 and Group 2

	Total(n = 125)	Group 1(n = 69)	Group 2(n = 56)	*P*
Age (y)	68.8 ± 6.7	67.9 ± 6.3	69.8 ± 7.0	0.132
Sex (female)	92 (73.6)	51 (73.9)	41 (73.2)	0.93
Alcohol	8 (6.8)	5 (7.8)	3 (5.6)	0.668
Smoke	6 (5.0)	5 (7.8)	1 (1.8)	0.156
BUN (mmol/L)	5.77 ± 1.61	5.87 ± 1.37	5.64 ± 1.88	0.454
eGFR (ml/min)	84.87 ± 13.70	85.32 ± 13.94	84.32 ± 13.52	0.694
Scr (umol/L)	68.32 ± 15.4	68.30 ± 13.40	68.35 ± 17.71	0.985
SUA (umol/L)	318.68 ± 78.04	322.88 ± 74.83	313.45 ± 82.29	0.515
ALT (U/L)	19.25 ± 12.21	18.49 ± 11.15	20.20 ± 13.48	0.449
AST (U/L)	19.77 ± 9.16	19.47 ± 8.30	20.14 ± 10.18	0.693
ALP (U/L)	71.07 ± 22.15	71.52 ± 25.35	70.53 ± 17.84	0.812
TC (mmol/L)	4.74 ± 0.89	4.68 ± 0.87	4.81 ± 0.92	0.487
TG (mmol/L)	1.56 ± 0.77	1.40 ± 0.62	1.77 ± 0.90	0.021^*^
HDL (mmol/L)	1.50 ± 0.38	1.47 ± 0.43	1.54 ± 0.31	0.417
LDL (mmol/L)	2.85 ± 0.80	2.79 ± 0.76	2.95 ± 0.86	0.376
HbA1c (%)	5.95 ± 0.64	5.86 ± 0.50	6.06 ± 0.79	0.128

^*^P < 0.05. P values were 2-sided and values < 0.05 were considered to be significantly important

**Fig. 3:**
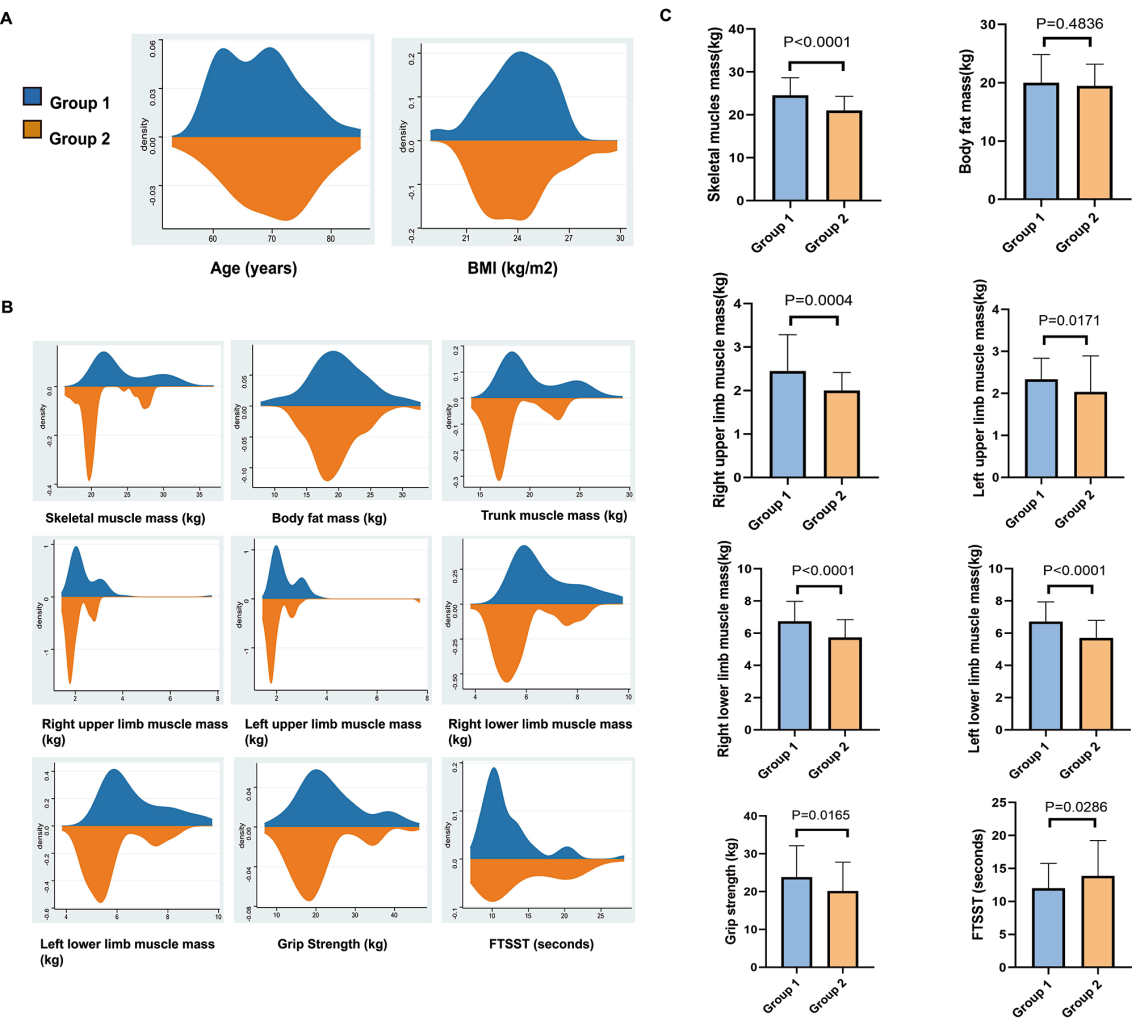
(**A**-**B**) Differences in age, body composition and skeletal muscle function between Group 1 and Group 2. (**C**) Bar charts of body composition and skeletal muscle function, presented as the mean ± standard deviation. P values were 2-sided, and values < 0.05 were considered to be significantly important

Table 2; Fig. 3 display the body composition, skeletal muscle function and BMD characteristics. Differences in BMI and body fat mass were not significant between the 2 groups. However, Group 2 exhibited lower levels of body composition parameters, including SMM, muscle mass of four extremities, trunk muscle mass, ASM, SMI, and basal metabolic rate, compared to Group 1 (*P* < 0.05). Individuals in the 2 groups exhibited differences in grip strength and FTTST. Group 1 patients performed better in grip strength and FTTST than Group 2 patients. Specifically, grip strength (*P =* 0.017) was decreased, and FTTST was increased (*P =* 0.029) in Group 2 in comparison with Group 1. BMD and the numbers of individuals with osteopenia or osteoporosis were significantly different between the 2 groups. Except for lumbar spine BMD (*P =* 0.833), femoral neck BMD (*P =* 0.011) and total hip BMD (*P =* 0.043) of Group 2 individuals were decreased compared to Group (1) Out of the 40 individuals with osteoporosis, 17 belonged to Group 1, and 23 belonged to Group (2) Moreover, out of the 106 individuals diagnosed with osteopenia or osteoporosis, 53 were in Group 1, and 53 were in Group 2. The number of individuals diagnosed with osteopenia and osteoporosis in Group 2 dramatically increased compared with Group 1 (*P* = 0.006).

**Table 2:** Basic information, body composition, skeletal muscle function and BMD of Group 1 and Group 2

	Total(n = 125)	Group 1(n = 69)	Group 2(n = 56)	*P*
Height (cm)	161.0 ± 7.8	164.2 ± 7.1	157.1 ± 6.9	< 0.001^***^
Weight (kg)	62.0 ± 7.9	64.7 ± 8.2	58.7 ± 6.2	< 0.001^***^
BMI (kg/m^2^)	24.0 ± 1.9	23.9 ± 1.8	24.1 ± 2.1	0.599
Body fat mass (kg)	19.8 ± 4.4	20.0 ± 4.8	19.5 ± 3.7	0.484
SMM (kg)	23.0 ± 4.2	24.6 ± 4.1	21.0 ± 3.3	< 0.001^***^
Right upper limb muscle mass (kg)	2.2 ± 0.7	2.5 ± 0.8	2.0 ± 0.4	< 0.001^***^
Left upper limb muscle mass (kg)	2.2 ± 0.7	2.3 ± 0.5	2.0 ± 0.9	0.017^*^
Trunk muscle mass (kg)	19.2 ± 3.2	20.4 ± 3.2	17.8 ± 2.6	< 0.001^***^
Right lower limb muscle mass (kg)	6.3 ± 1.3	6.7 ± 1.2	5.7 ± 1.1	< 0.001^***^
Left lower limb muscle mass (kg)	6.3 ± 1.3	6.7 ± 1.2	5.7 ± 1.1	< 0.001^***^
ASM (kg)	17.0 ± 3.6	18.2 ± 3.5	15.5 ± 3.1	< 0.001^***^
SMI (kg/m^2^)	6.49 ± 0.83	6.71 ± 0.80	6.21 ± 0.78	0.001^**^
Basal metabolic rate (kcal)	1288.8 ± 148.7	1346.5 ± 146.2	1217.8 ± 118.9	< 0.001^***^
Grip strength (kg)	22.3 ± 8.2	23.9 ± 8.3	20.2 ± 7.6	0.017^*^
FTSST (s)	12.82 ± 4.61	11.99 ± 3.78	13.89 ± 5.35	0.029^*^
Lumbar spine BMD (g/cm^2^)	0.891 ± 0.183	0.894 ± 0.187	0.887 ± 0.189	0.833
Total hip BMD (g/cm^2^)	0.796 ± 0.138	0.819 ± 0.139	0.768 ± 0.131	0.043^*^
Femoral neck BMD (g/cm^2^)	0.656 ± 0.115	0.679 ± 0.124	0.626 ± 0.096	0.011^*^
Osteoporosis	40 (32.0)	17 (24.6)	23 (41.1)	0.05
Osteopenia & Osteoporosis	106 (84.8)	53 (76.8)	53 (94.6)	0.006^*^

^*^P < 0.05; ^**^P < 0.01; ^***^P < 0.001. P values were 2-sided and values < 0.05 were considered to be significantly important

As shown in Table 3, IL-6 (*P* = 0.049) and CRP (*P* < 0.001), 2 inflammatory markers, were found to be different between the 2 groups. The expression levels of IL-6 and CRP in Group 1 were significantly decreased compared with those in Group 2. Then, we examined the correlation between IL-6 and CRP and various body parameters, including body fat mass, grip strength, SMM, lumbar spine BMD, total hip BMD and femoral neck BMD. IL-6 was significantly associated with all of the aforementioned parameters. Specifically, IL-6 was positively correlated with body fat mass (*P* = 0.013) and negatively correlated with grip strength (*P* = 0.008), SMM (*P* = 0.029) and BMD values. On the other hand, CRP was only negatively correlated with BMD values and had no correlation with body fat mass, grip strength or SMM.

**Table 3:** The levels of IL-6 and CRP and the correlation between IL-6 and CRP and parameters of fat, skeletal muscle and BMD.

	Total(n = 125)	Group 1(n = 69)	Group 2(n = 56)	*P*
IL-6 (pg/mL)	4.23 ± 5.49	2.89 ± 1.33	3.10 ± 7.48	0.049^*^
CRP (mg/L)	0.62 ± 0.19	0.49 ± 0.09	0.74 ± 0.17	< 0.001^***^
	**IL-6**		**CRP**	
Body fat mass	0.238 (0.013)^*^		0.14 (0.089)	
Grip strength	-0.394 (0.008)^*^		-0.12 (0.266)	
SMM	-0.184 (0.029)^*^		-0.144 (0.075)	
Lumbar spine BMD	-0.336 (0.004)^**^		-0.651 (< 0.001)^***^	
Total hip BMD	-0.439 (< 0.001)^***^		-0.823 (< 0.001)^***^	
Femoral neck BMD	-0.356 (0.002)^**^		-0.651 (< 0.001)^***^	

^*^P < 0.05; ^**^P < 0.01; ^***^P < 0.001. P values were 2-sided and values < 0.05 were considered to be significantly important. The upper half of the table displays the expression levels and intergroup differences of IL-6 and CRP. In the bottom half of the table, numbers outside parentheses are Spearman correlation coefficients and numbers inside parentheses are *P*-values

**Fig. 4:**
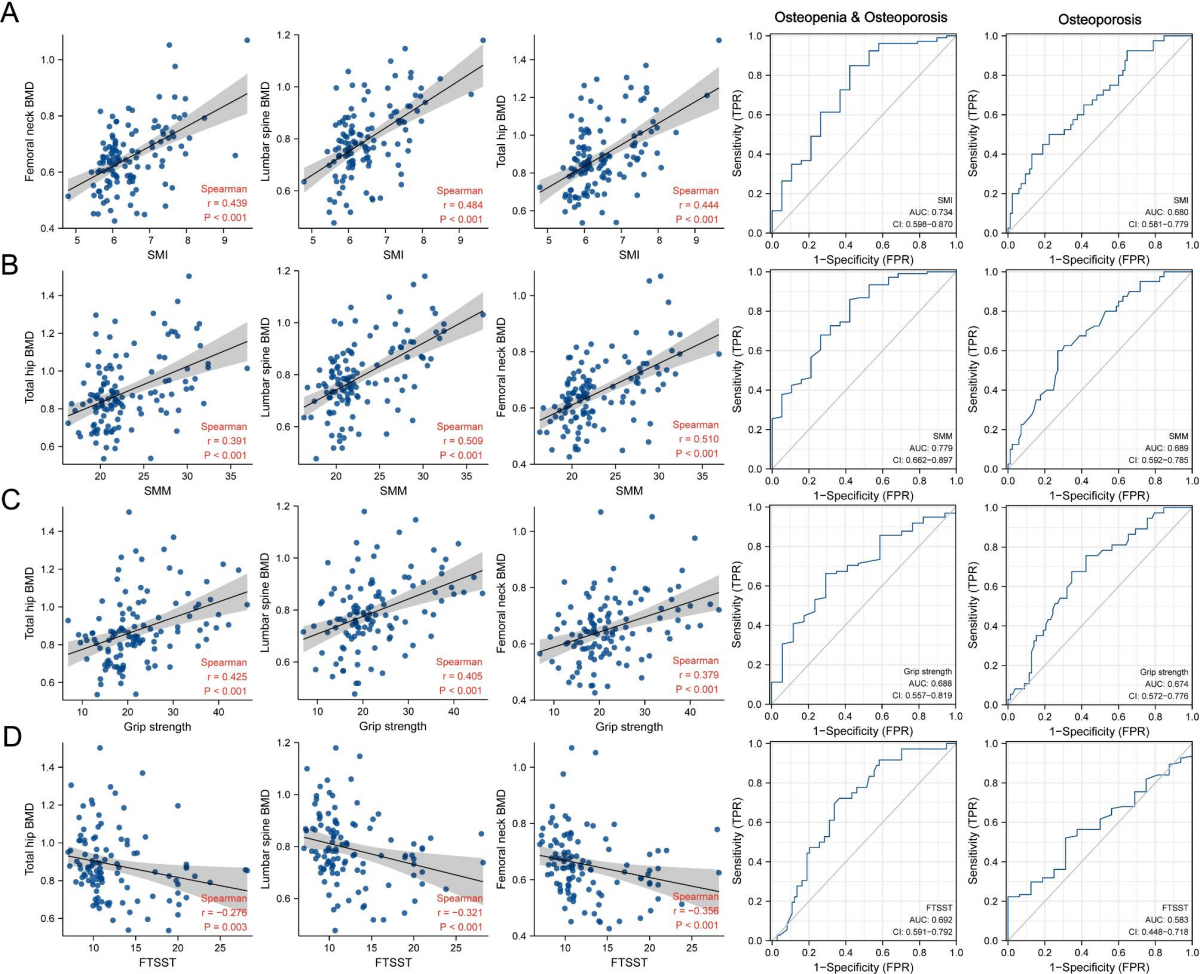
(**A**) Scatter plots show the correlation between skeletal muscle parameters and BMD. The gray part indicates the 95% confidence interval. The ROC curves represent the performance of the prediction of osteopenia or osteoporosis through SMI. (**B**) SMM. (**C**) Grip strength. (**D**) FTTST. P values were 2-sided, and values < 0.05 were considered to be significantly important. Abbreviations: SMI, skeletal muscle mass index; BMD, bone mineral density; ROC, receiver operating characteristic; FTTST, 5-times sit-to-stand test

We also analyzed the correlation between skeletal muscle content (SMM and SMI), skeletal muscle function (grip strength and FTTST), and BMD in all 125 individuals. Our findings, represented in Fig. 4, indicate a significant correlation between skeletal muscle content, skeletal muscle function, and BMD (*P* < 0.01). Specifically, we observed positive correlations between SMM, SMI, grip strength, and BMD, while negative correlations were noted between FTTST and BMD.

### Bioinformatics analysis revealed immune homeostasis and adipokines associated with skeletal muscle function

**Fig. 5:**
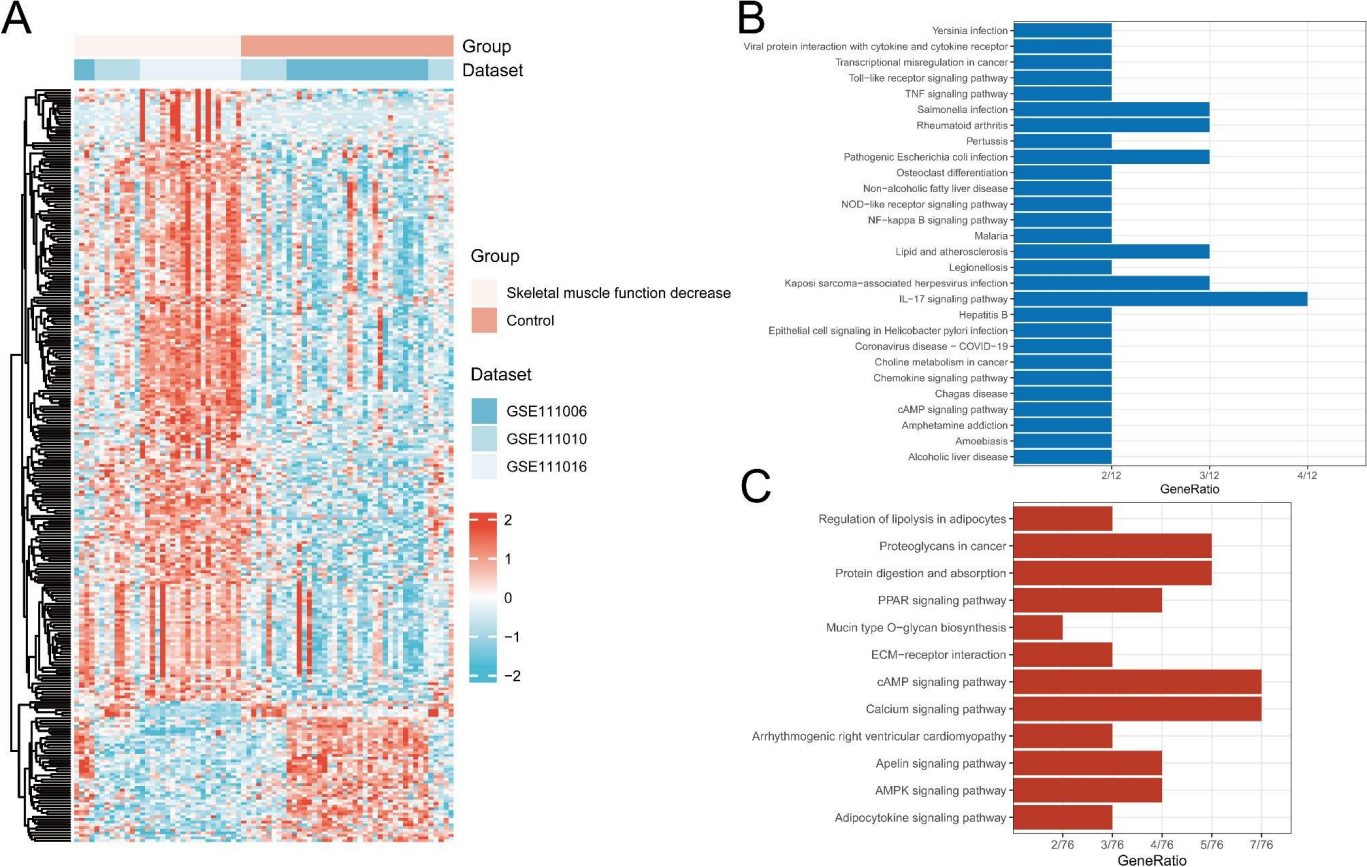
(**A**) Heatmap of DEGs. (**B**) KEGG analysis based on downregulated DEGs. (**C**) KEGG analysis based on upregulated DEGs. Abbreviation: DEGs, differentially expressed genes; KEGG, Kyoto Encyclopedia of Genes and Genomes

We retrospectively analyzed 33 samples with decreased skeletal muscle function and 42 control samples from GSE111006, GSE111010, and GSE111016. A total of 283 DEGs were detected, among which 230 showed upregulation, whereas 53 displayed downregulation. The heatmap in Fig. 5A demonstrates that these 2 groups were significantly different. More details of these genes can be found in Supplementary File 2. To obtain a preliminary understanding of these DEGs, we conducted KEGG enrichment for both upregulated and downregulated DEGs separately. The top 27 downregulated DEG-enriched notable KEGG pathways and the top 12 upregulated DEG-enriched KEGG pathways were demonstrated. These pathways were related to various biological processes, such as the immune response, bone metabolism, inflammation, and lipids (Fig. 5B-C).

Twenty-six IRGs were identified by screening DEGs using the ImmPort database. These IRGs included *CRHR2*, *ACKR2*, *ADIPOQ*, *FGF18*, *ANGPTL5*, *ELN*, *PLA2G2A*, *SEMA3D*, *RBP4*, *PTGER3*, *PTHLH*, *FGF7P3*, *CCL21*, *ANGPTL7*, *CXCL2*, *AZGP1*, *S100B*, *RORB*, *LEP*, *MARCO*, *CCL13*, *LHCGR*, *OGN*, *FOS*, *BMP7*, and *CXCL8*. Details of these genes are provided in Supplementary File 3. Notably, ADIPOQ, LEP, and RBP4 were involved in lipid metabolism. Additionally, these 3 genes were all overexpressed in samples with decreased skeletal muscle function compared to control samples.

**Fig. 6:**
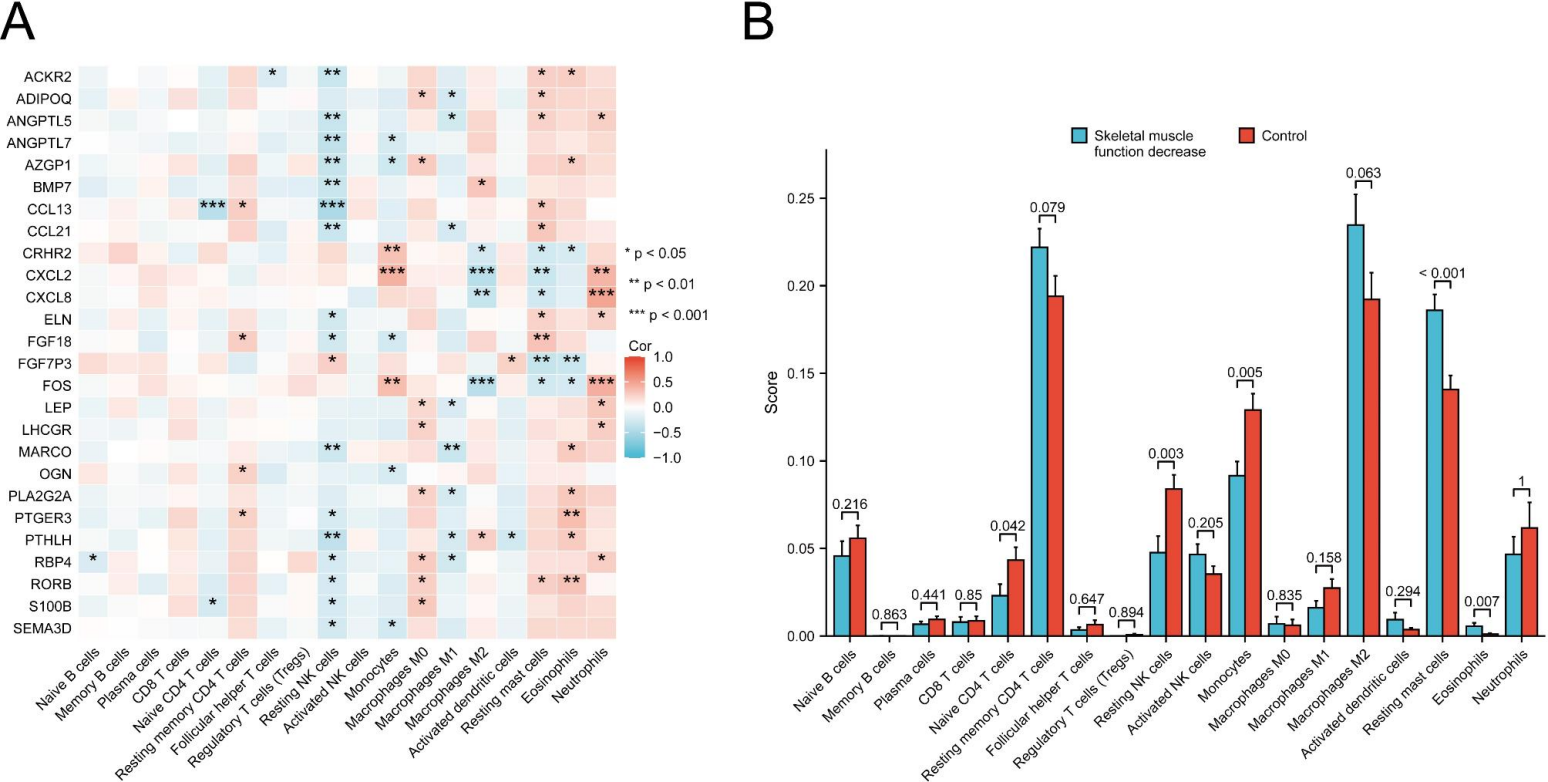
(**A**) Correlation heatmap of immune cells and IRGs. Red indicates a positive correlation, while blue indicates a negative correlation. (**B**) Grouped comparison histogram of immune infiltration. *P < 0.05; **P < 0.01. *P* values were 2-sided, and values < 0.05 were considered to be significantly important. Abbreviations: IRGs, immune-related genes

The CIBETSORT algorithm was utilized to evaluate immune cell infiltration associated with skeletal muscle function. The correlation between IRGs and immune cell infiltration was analyzed (Fig. 6). We observed a significantly lower proportion of naïve CD4 T cells in the skeletal muscle function decrease samples than in the control samples (*P* = 0.042). Furthermore, we found a significantly higher proportion of resting NK cells in the skeletal muscle function decrease samples (*P* = 0.003). Additionally, the proportion of monocytes was significantly reduced in the skeletal muscle function decrease samples (*P* = 0.005). The study found that the proportions of monocytes (*P* = 0.005) and resting mast cells (*P* < 0.001) significantly declined in samples with decreased skeletal muscle function, while the proportion of eosinophils increased significantly (*P* = 0.007). The study also found a universal correlation between NK cells, monocytes, macrophages, resting mast cells, eosinophils, neutrophils, and IRGs.

### LEP, ADIPOQ, RBP4, DPP4 and CXCL8 are potential meditators between skeletal muscle function and BMD

**Fig. 7:**
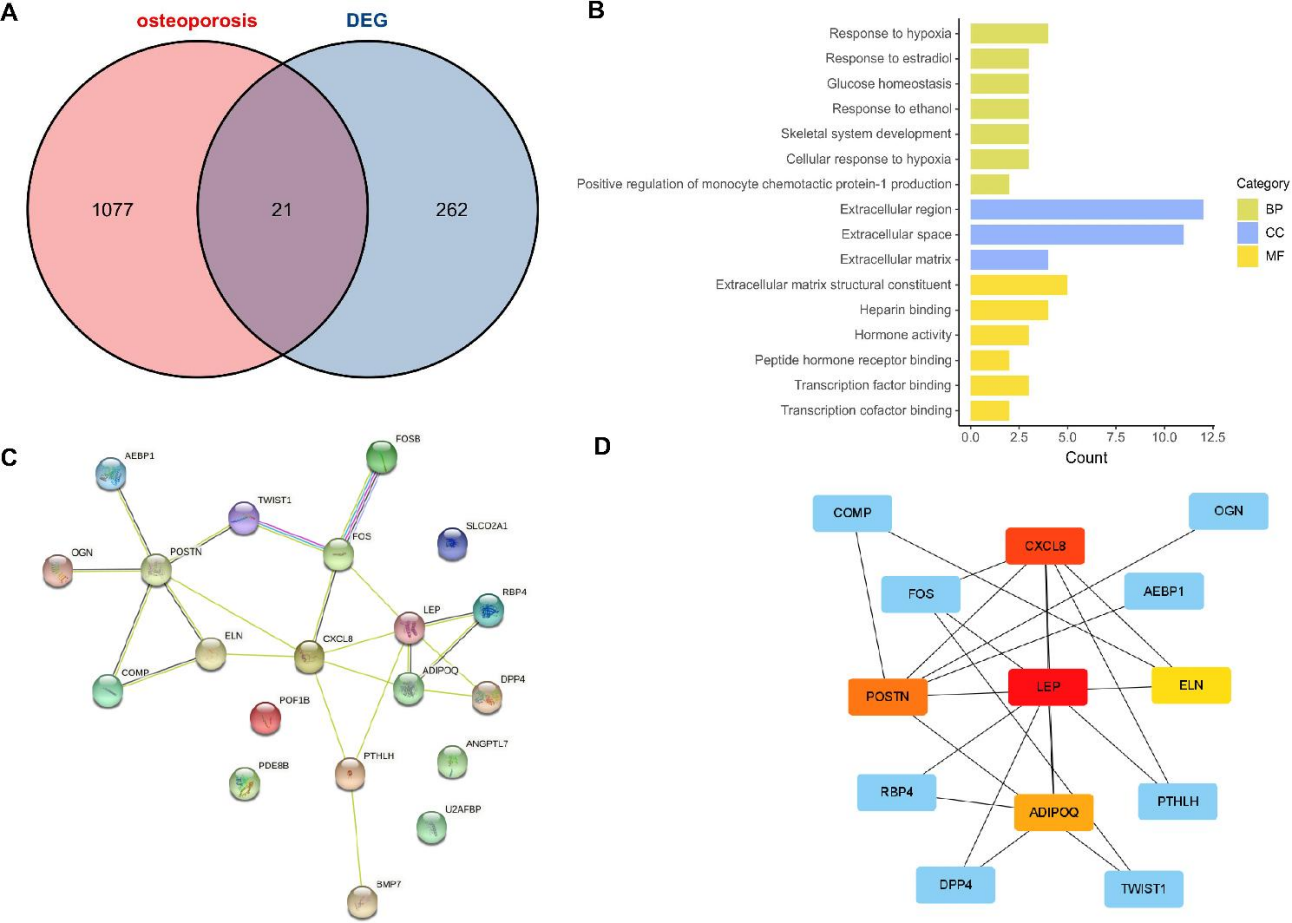
(**A**) Intersection of DEGs and osteoporosis-related genes in the DisGeNET database. (**B**) GO analysis of common targets between deceased skeletal muscle function and osteoporosis. (**C**) PPI network of common targets. Confidence score: > 0.4. (**D**) Major common targets in PPI work. Abbreviations: DEGs, differentially expressed genes; GO, Gene Ontology; PPI, protein–protein interaction; BP, biological process; CC, cellular component; MF, molecular function

Twenty-one common targets were identified between skeletal muscle function and BMD (Fig. 7A). These targets included *ADIPOQ*, *AEBP1*, *ANGPTL7*, *BMP7*, *COMP*, *CXCL8*, *DPP4*, *ELN*, *FOS*, *FOSB*, *LEP*, *LINC00311*, *OGN*, *PDE8B*, *POF1B*, *POSTN*, *PTHLH*, *RBP4*, *SLCO2A1*, *TWIST1*, and *U2AF1*. Their biological process (BP), molecular function (MF), and cellular component (CC) are shown in Fig. 7B. The STRING database was utilized to construct the PPI network for these genes, with a target being excluded due to missing data. This PPI network consisted of 20 nodes and 22 edges, and the mean node degree and mean local clustering coefficient were 2.2 and 0.47, respectively. As illustrated in Fig. 7C, the *P* value of PPI enrichment was 1.02e-09. We further analyzed the targets in the PPI network using Cytoscape 3.8.0 software (Fig. 7D). We identified 4 adipokine targets, LEP, ADIPOQ, RBP4 and DPP4, that were closely related in the network. In addition, CXCL8 was observed to be associated with these adipokines.

### The expression levels of LEP, ADIPOQ, RBP4, DPP4, CXCL8 and TNF-α were relatively increased in individuals in Group 2 compared to those in Group 1

**Fig. 8:**
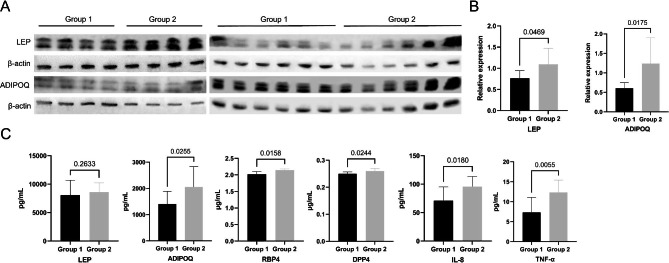
(**A**) The expression of LEP and ADIPOQ in PBMCs of included participants in Group 1 (n = 10) and Group 2 (n = 10). β-actin was used as the internal reference gene. (**B**) Relative expression of LEP and ADIPOQ in PBMCs measured by WB (n = 10 each). (**C**) The plasma levels of LEP, ADIPOQ, RBP4, DPP4, IL-8 and TNF-α were measured by ELISA in Group 1 (n = 12) and Group 2 (n = 12). P values were 2-sided, and values < 0.05 were considered to be significantly important. Abbreviations: LEP, leptin; ADIPOQ, adiponectin; SMM, skeletal muscle mass; PBMCs, peripheral blood mononuclear cells; WB, western blotting; RBP4, retinol binding protein 4; DPP4, dipeptidyl peptidase 4; IL-8, interleukin-8; TNF-α, tumor necrosis factor-α

Ten individuals were selected from each group, and their whole blood was extracted to isolate PBMCs. LEP and ADIPOQ levels within PBMCs were measured using WB. As shown in Fig. 8A-B, LEP (*P* = 0.0469) and ADIPOQ (*P* = 0.0175) expression was relatively upregulated in the PBMCs of Group 2 individuals in comparison with Group 1 individuals. In addition, blood samples were collected from 12 individuals in each group and separated into plasma. We assessed the expression levels of LEP, ADIPOQ, RBP4, DPP4, CXCL8 (also known as IL-8) and TNF-α in the plasma of both groups using ELISA. According to Fig. 8C, while the difference in plasma LEP level was not significant (*P* = 0.2633) between groups, Group 2 individuals displayed apparently increased plasma ADIPOQ (*P* = 0.0255), RBP4 (*P* = 0.0158), DPP4 (*P* = 0.0244), IL-8 (*P* = 0.018) and TNF-α (*P* = 0.0055) levels than Group 1 individuals. It was found that there was relative consistency in the expression levels of adipokines in both PBMCs and plasma among middle-aged and elderly individuals in this cross-sectional study, thereby indicating that adipokine expression may be crucial for skeletal muscle function and BMD.

## Discussion

Both sarcopenia and osteoporosis are linked with numerous negative outcomes, including fractures, falls, hospitalization or disability [16]. Given the global aging population, it is important to investigate the mechanism between skeletal muscle function and BMD to establish effective clinical decision-making strategies. Previous research has demonstrated a relationship between inflammation, skeletal muscle and BMD [2, 17–19]. The objective of this study was to explore the potential correlation between adipokines, skeletal muscle function and BMD. The results revealed a significant correlation between these factors. Middle-aged and elderly individuals with lower skeletal muscle function and BMD had higher expression levels of LEP, ADIPOQ, RBP4, DPP4 and inflammatory markers, suggesting that adipokines may play a role in the activation of inflammation and lead to the development of sarcopenia and osteoporosis.

Our cross-sectional study found a significant association between skeletal muscle content, skeletal muscle function and BMD. Specifically, higher levels of SMM and worse skeletal muscle function were related to decreased BMD and a higher osteoporosis risk. These findings support the notion that aging skeletal muscle is strongly linked to osteoporosis, which is mentioned in some articles. Simon et al. investigated the relationship between skeletal muscle function and bone microarchitecture in females. They discovered a significant correlation between skeletal muscle strength, cortical bone thickness, and bone cortical area, highlighting the importance of assessing muscle function in evaluating fracture risk [20]. Kuriyama et al. examined the correlation between skeletal muscle and osteoporosis among middle-aged and old people in Japan and found that male subjects with lower SMM were more likely to develop osteoporosis [21].

The relationships between inflammatory markers (IL-6, IL-8, CRP and TNF-α) and various parameters related to fat, skeletal muscle and BMD were also investigated. Our findings revealed that higher levels of inflammatory markers are generally associated with increased body fat mass, decreased grip strength, decreased SMM and higher BMD in these individuals, which aligned with the observed expression condition of adipokines. IL-6, working as a pro-inflammatory factor, can selectively stimulate protein degradation and promote fat synthesis in human skeletal muscle [22]. TNF-α is secreted by both adipose tissue and skeletal muscle, and its presence can hinder muscle protein synthesis by promoting insulin resistance [23]. In addition, IL-6 and TNF-α can enhance the bone resorption capacity of PBMCs, stimulate the formation of osteoclasts and contribute to the development of osteoporosis [24]. IL-8 and CRP are nonspecific inflammatory markers. Several clinical studies have established a significant correlation between elevated levels of IL-8 and CRP and conditions such as obesity, sarcopenia, and osteoporosis [25]. However, CRP in this study was not correlated with body fat mass, grip strength or SMM, which may be attributable to its lower sensitivity in predicting fat or skeletal muscle compared with IL-6 [26, 27].

By utilizing bioinformatics methods, we identified that LEP, ADIPQO, RBP4 and DPP4 are associated with decreased skeletal muscle function. This discovery reinforces the conclusion that increased adipokine levels may have adverse effects on the physiological function of skeletal muscles [28, 29]. Relationships between adipokines, skeletal muscle and bone metabolism are intricate. Leptin and adiponectin work together to maintain muscular function. Leptin is produced by subcutaneous fat and skeletal muscle [30] and activates the AMPK/ERK pathway to regulate protein metabolism in skeletal muscle [31]. Leptin has a bidirectional regulatory effect on bone, affecting both anabolism and catabolism, with its effects influenced by the presence of a central relay. Moreover, leptin has been shown to have proinflammatory effects and to promote the generation of proinflammatory factors such as IL-6 and TNF-α in macrophages and monocytes [32]. On the other hand, adiponectin is mainly secreted [33] by brown adipose tissue, which exerts an essential function in promoting mitochondrial synthesis and activating the AMP-activated protein kinase (AMPK) pathway [34]. Adiponectin has been found to increase extracellular calcium influx via adiponectin receptor 1 (AdipoR1), thus upregulating peroxisome proliferator-activated receptor gamma coactivator-1α (PGC-1α) and mitochondria in muscle cells [35]. However, clinical trials have shown that adiponectin has a negative relationship with BMD in obese humans [36–38]. In a human coculture system containing PBMCs, adiponectin can promote the differentiation of osteoclasts in an osteoblast-dependent manner [39]. Additionally, a study showed that high-molecular-weight adiponectin can enhance IL-8 translation by human macrophages [40], which is consistent with our analysis of the PPI network.

RBP4 is a cytokine that is secreted by adipose tissue and has proinflammatory properties. Studies have shown that RBP4 can lead to insulin resistance and affect protein anabolism in skeletal muscle indirectly, particularly under the stimulation of peroxisome proliferator-activated receptor-gamma (PPARγ) agonists [41]. Additionally, RBP4 has been found to activate adipose tissue macrophages and prime the NLRP3 inflammasome to produce IL-1β, IL-6 and TNF-α through Toll-like receptors 2/4 [42, 43]. A clinical study has shown that circulating RBP4 levels are closely related to SMM and muscle function among elderly individuals, making it a useful biomarker for screening sarcopenia [44]. DPP4 was identified as another adipokine that could be linked to skeletal muscle function and BMD in this study. DPP4 can produce IL-6 and TNF-α [45, 46] and exacerbate inflammation mediated by adipose tissue macrophages by interacting with adenosine deaminase (ADA) and other extracellular matrix proteins [47]. DPP4 can also impair the insulin signaling pathway, thereby contributing to insulin resistance [47]. Recent research has reported that elevated levels of DPP4 in elderly diabetes patients possibly induce the reduced mass and function of skeletal muscle [48, 49]. In addition, DPP4 has obvious negative effects on bone tissue. Zhang et al. discovered that higher levels of DPP4 are related to low BMD in postmenopausal women as well as a higher osteoporosis risk [50].

### Study strengths and limitations

This study has several advantages. Samples assigned to 2 groups in the cross-sectional study were middle-aged and elderly individuals at similar ages, thus avoiding the interference of natural aging on skeletal muscles to the maximum extent. This study examined the correlation between BMD and skeletal muscle function from the perspective of adipokines and suggests that adipokines could be the key factors responsible for the degeneration of bone and skeletal muscle. Notably, our findings reveal for the first time that the protein levels of leptin and adiponectin within PBMCs can impact muscle mass and function. These clinically relevant findings could pave the way for developing new treatments to elevate skeletal muscle function in the future. This study, however, has notable shortcomings. Our clinical study had a small sample size, which has limited the ability to adjust for potential confounding factors. For instance, although there were differences in TG levels between the two groups, our study did not find any correlation between TG and skeletal muscle parameters or BMD. In addition, no follow-up was performed in this study, and future studies should incorporate a follow-up period to clarify the long-term impact of adipokines on skeletal muscle function. Moreover, we did not perform in vivo experiments to validate our conclusion.

## Conclusions

Our clinical study and in vitro experiments have shown that individuals with decreased skeletal muscle function exhibit increased expression of LEP, ADIPOQ, RBP4, DPP4 and inflammatory markers. The increased levels of adipokines in skeletal muscle are possibly associated with a decrease in BMD and the occurrence of osteoporosis among middle-aged and elderly individuals. More research on the implications of this relationship and the development of effective clinical treatments are needed.

## Electronic supplementary material

Below is the link to the electronic supplementary material.

Supplementary Material 1

## Data Availability

Data used in this work can be obtained from the corresponding author upon request.

## Abbreviations

SMM: Skeletal muscle mass
SMI: Skeletal muscle mass index
IMCL: Intramyocellular lipids
PBMCs: Peripheral blood mononuclear cells
BMD: Bone mineral density
WB: Western blotting
ALT: Alanine aminotransferase
WHO: World Health Organization
eGFR: Estimated glomerular filtration rate
Scr: Serum creatinine
BUN: Blood urea nitrogen
SUA: Serum uric acid
AST: Aspartate transaminase
ALP: Alkaline phosphatase
TG: Triglycerides
TC: Total cholesterol
HDL: High-density lipoprotein
LDL: Low-density lipoprotein
HbA1c: Glycated hemoglobin
CRP: C-reactive protein
GEO: Gene Expression Omnibus
DEGs: Differentially expressed genes
KEGG: Kyoto Encyclopedia of Genes and Genomes
IRGs: Immune-related genes
PCA: Principal component analysis
ROC: Receiver operating characteristic
AUC: Area under the curve
PRC: Precision-recall curve
GO: Gene ontology
PPI: Protein‒protein interaction
ASM: Appendicular skeletal muscle mass
FTSST: 5-Times Sit-to-Stand Test
HRP: Avidin-Horseradish Peroxidase
BP: Biological process
MF: Molecular function
CC: Cellular component
AMPK: AMP-activated protein kinase
PGC-1α: Peroxisome proliferator-activated receptor gamma coactivator-1α
AdipoR1: Adiponectin receptor 1
PPARγ: Peroxisome proliferator-activated receptor-gamma
ADA: Adenosine deaminase
