# Supplementary material for: A potential correlation between adipokines, skeletal muscle function and bone mineral density in middle-aged and elderly individuals
Source: Lipids Health Dis. 2023 Jul 31;22:111. doi: 10.1186/s12944-023-01879-z (PMC10388529; doi:10.1186/s12944-023-01879-z)
Supplement: Supplementary file 1 — Supplementary Material 1 [file 12944_2023_1879_MOESM1_ESM.docx]

**Supplementary File 1**

The correlation coefficients and P value between TG levels and parameters of skeletal muscle or BMD values in participants by Spearman’s correlation analysis.

| Parameters | TG: r (P-value) |
| --- | --- |
| Skeletal muscle mass | -0.051 (0.654) |
| Right upper limb muscle mass | -0.01 (0.931) |
| Left upper limb muscle mass | -0.035 (0.756) |
| Trunk muscle mass | -0.028 (0.803) |
| Right lower limb muscle mass | -0.055 (0.633) |
| Left lower limb muscle mass | -0.034 (0.766) |
| ASM | -0.045 (0.692) |
| SMI | -0.061 (0.591) |
| Total hip BMD | 0.196 (0.083) |
| Lumbar spine BMD | 0.151 (0.185) |
| Femoral neck BMD | 0.212 (0.061) |
| FTSST | 0.068 (0.553) |
| Grip strength | -0.076 (0.508) |

Abbreviations: TG, triglycerides; BMD, bone mineral density. Numbers outside parentheses are Spearman correlation coefficients and numbers inside parentheses are P values.

The correlation coefficients and P value between HbA1c levels and parameters of skeletal muscle or BMD values in participants by Spearman’s correlation analysis.

| Parameters | HbA1c: r (P-value) |
| --- | --- |
| Skeletal muscle mass | -0.019 (0.863) |
| Right upper limb muscle mass | 0.041 (0.704) |
| Left upper limb muscle mass | 0.04 (0.712) |
| Trunk muscle mass | 0.022 (0.842) |
| Right lower limb muscle mass | -0.069 (0.529) |
| Left lower limb muscle mass | -0.075 (0.491) |
| ASM | -0.036 (0.742) |
| SMI | -0.008 (0.942) |
| Total hip BMD | -0.098 (0.370) |
| Lumbar spine BMD | -0.068 (0.536) |
| Femoral neck BMD | -0.099 (0.364) |
| FTSST | 0.111 (0.307) |
| Grip strength | -0.128 (0.241) |

Abbreviations: BMD, bone mineral density. Numbers outside parentheses are Spearman correlation coefficients and numbers inside parentheses are P values.

**Supplementary File 2**

Differentially expressed genes of decreased skeletal muscle function.

| Gene name | logFC | P.Value | adj.P.Val |
| --- | --- | --- | --- |
| COX20P1 | -1.144150784 | 9.49E-12 | 3.66E-07 |
| RPS3AP38 | 1.040073976 | 1.58E-10 | 2.07E-06 |
| LVRN | 1.358275686 | 3.24E-10 | 3.13E-06 |
| PCK1 | 3.104456464 | 4.22E-10 | 3.26E-06 |
| MIR550A3 | 1.394297276 | 8.23E-10 | 4.44E-06 |
| COL8A2 | 1.485378375 | 9.21E-10 | 4.44E-06 |
| GPRC5A | 1.451859866 | 1.20E-09 | 5.05E-06 |
| PCOLCE2 | 1.840706013 | 1.37E-09 | 5.05E-06 |
| ABI3BP | 1.114672371 | 1.44E-09 | 5.05E-06 |
| DNM1 | 1.096302244 | 1.67E-09 | 5.36E-06 |
| PRG4 | 2.165017836 | 2.27E-09 | 6.74E-06 |
| NOVA1 | 1.156302288 | 3.43E-09 | 7.79E-06 |
| LINC00632 | 1.363188243 | 4.87E-09 | 9.70E-06 |
| COMP | 3.607429277 | 5.03E-09 | 9.70E-06 |
| THBS2 | 1.307217303 | 5.77E-09 | 1.06E-05 |
| FBLN1 | 1.113938584 | 6.64E-09 | 1.12E-05 |
| EFEMP1 | 1.299711873 | 7.57E-09 | 1.17E-05 |
| COL14A1 | 1.347005494 | 1.31E-08 | 1.63E-05 |
| ANGPTL8 | 1.802681999 | 1.70E-08 | 1.87E-05 |
| LINC00222 | 1.025230876 | 1.80E-08 | 1.93E-05 |
| GFPT2 | 1.08330826 | 2.29E-08 | 2.33E-05 |
| ZNF559-ZNF177 | 1.23286176 | 2.52E-08 | 2.38E-05 |
| RPLP0P2 | -1.116837344 | 2.78E-08 | 2.55E-05 |
| RNY4 | 1.471148589 | 3.08E-08 | 2.76E-05 |
| CDS1 | 1.495605281 | 3.40E-08 | 2.89E-05 |
| MTRNR2L1 | -2.278130646 | 5.12E-08 | 3.22E-05 |
| CRHR2 | -1.336290089 | 5.19E-08 | 3.22E-05 |
| MCOLN3 | 1.430688538 | 5.46E-08 | 3.24E-05 |
| HCAR1 | 1.962781442 | 5.55E-08 | 3.25E-05 |
| RNU4-13P | 1.402707493 | 7.00E-08 | 3.48E-05 |
| ITGA11 | 1.020411652 | 7.35E-08 | 3.48E-05 |
| ACKR2 | 1.03319462 | 9.00E-08 | 3.95E-05 |
| MT-RNR2 | -1.308694889 | 9.43E-08 | 4.00E-05 |
| CCT4P2 | 1.172326759 | 1.03E-07 | 4.22E-05 |
| PRELP | 1.082192768 | 1.09E-07 | 4.35E-05 |
| RNY3 | 1.060825138 | 1.23E-07 | 4.65E-05 |
| CIDEA | 2.371531456 | 1.28E-07 | 4.65E-05 |
| ADIPOQ | 3.267689249 | 1.42E-07 | 4.90E-05 |
| MIR1245A | 1.214670034 | 1.48E-07 | 4.98E-05 |
| FGF18 | 1.155269848 | 1.60E-07 | 5.11E-05 |
| RNY3P1 | 1.002020953 | 1.99E-07 | 5.69E-05 |
| C7 | 1.98440562 | 2.27E-07 | 6.13E-05 |
| PPP1R1B | 1.743677677 | 2.27E-07 | 6.13E-05 |
| MIR2355 | 1.13294545 | 2.29E-07 | 6.13E-05 |
| GALNT8 | 1.03193723 | 2.53E-07 | 6.60E-05 |
| FBN1 | 1.002687993 | 2.71E-07 | 6.79E-05 |
| ANGPTL5 | 1.360148871 | 2.89E-07 | 6.88E-05 |
| RPSAP6 | 1.137904864 | 2.93E-07 | 6.89E-05 |
| PPIAP10 | 1.014626251 | 3.14E-07 | 7.24E-05 |
| WNT2 | 1.19007145 | 4.02E-07 | 7.65E-05 |
| C6 | 1.168298557 | 4.46E-07 | 8.04E-05 |
| FAM180B | 1.05366381 | 4.55E-07 | 8.15E-05 |
| PIEZO2 | 1.245550926 | 4.66E-07 | 8.20E-05 |
| MKX | 1.860428349 | 4.95E-07 | 8.21E-05 |
| GABRE | 1.015744576 | 4.98E-07 | 8.21E-05 |
| EDIL3-DT | 1.226001081 | 5.11E-07 | 8.21E-05 |
| LMO3 | 1.549221643 | 5.27E-07 | 8.26E-05 |
| NDUFB10P2 | 1.036266948 | 5.76E-07 | 8.79E-05 |
| ELN | 1.037085818 | 6.23E-07 | 9.01E-05 |
| RPL21P135 | 1.09074854 | 6.40E-07 | 9.14E-05 |
| WNT10B | 1.260224483 | 7.21E-07 | 9.53E-05 |
| LYVE1 | 1.069801526 | 7.46E-07 | 9.69E-05 |
| PLA2G2A | 2.028693302 | 7.46E-07 | 9.69E-05 |
| EDIL3 | 1.546249955 | 7.92E-07 | 9.88E-05 |
| CADM3 | 1.220238576 | 8.26E-07 | 0.000100598 |
| MAL2 | 1.898213085 | 9.20E-07 | 0.000107568 |
| TNMD | 1.727893081 | 9.44E-07 | 0.00010844 |
| RN7SKP288 | 1.582752882 | 9.55E-07 | 0.000108779 |
| TTLL11-IT1 | 1.087558464 | 9.79E-07 | 0.000109598 |
| MIR199A2 | 1.002907778 | 1.00E-06 | 0.000111066 |
| CA12 | 1.599522371 | 1.17E-06 | 0.000121262 |
| LARGE-IT1 | 1.08540353 | 1.18E-06 | 0.00012205 |
| PDCL2P2 | -1.144370459 | 1.18E-06 | 0.00012205 |
| DPP4 | 1.064457984 | 1.35E-06 | 0.000129797 |
| SEMA3D | 1.566526563 | 1.42E-06 | 0.000134433 |
| RPL17P19 | 1.29997091 | 1.55E-06 | 0.000139744 |
| SFRP2 | 1.763728017 | 1.60E-06 | 0.000141539 |
| GLYAT | 1.775981683 | 1.61E-06 | 0.000141945 |
| DACT2 | 1.543384986 | 1.68E-06 | 0.000143872 |
| CLEC4G | 1.126666499 | 1.74E-06 | 0.000145902 |
| DDX3P2 | 1.00559403 | 1.78E-06 | 0.000147081 |
| C1QTNF7 | 1.063137267 | 1.91E-06 | 0.000149119 |
| VASH2 | 1.032589659 | 1.94E-06 | 0.000149978 |
| GPC3 | 1.319237592 | 1.98E-06 | 0.000151924 |
| AHNAK2 | 1.117574976 | 2.11E-06 | 0.000156928 |
| MIR5699 | 1.000401466 | 2.13E-06 | 0.000158366 |
| HUNK | 1.234394485 | 2.14E-06 | 0.000158573 |
| RBP4 | 2.271709403 | 2.31E-06 | 0.000163078 |
| SPON1 | 1.815297669 | 2.32E-06 | 0.000163078 |
| CHL1 | 1.391067464 | 2.33E-06 | 0.000163078 |
| ADGRG2 | 1.409602753 | 2.39E-06 | 0.00016528 |
| KLB | 2.214007477 | 2.61E-06 | 0.000173723 |
| PRRX2 | 1.400905227 | 2.82E-06 | 0.000181207 |
| NTRK2 | 1.389688001 | 2.88E-06 | 0.00018344 |
| GYG2 | 2.174083185 | 2.92E-06 | 0.000185426 |
| PTGER3 | 1.350000156 | 2.95E-06 | 0.000185744 |
| PTGES | 1.115787904 | 3.02E-06 | 0.00018793 |
| RAP2CP1 | -1.103041478 | 3.27E-06 | 0.000195798 |
| RNU6-322P | 1.175247043 | 3.30E-06 | 0.000196536 |
| AEBP1 | 1.020233279 | 3.33E-06 | 0.000196536 |
| CIDEC | 2.451833869 | 3.56E-06 | 0.000206153 |
| CYCSP28 | 1.026804981 | 3.70E-06 | 0.000209822 |
| CACNA1C-IT3 | 1.144896644 | 3.75E-06 | 0.00021125 |
| SHISAL1 | 1.30238525 | 4.17E-06 | 0.000222815 |
| RNU1-93P | 1.098715836 | 4.55E-06 | 0.000234914 |
| LINC00645 | 1.34813644 | 4.66E-06 | 0.000238745 |
| PTHLH | 1.199181716 | 5.53E-06 | 0.00026577 |
| PLIN1 | 1.912962011 | 5.65E-06 | 0.000268702 |
| FGF14-IT1 | 1.144960678 | 5.95E-06 | 0.000277159 |
| FGF7P3 | -1.189095221 | 6.15E-06 | 0.000279981 |
| RP1L1 | -1.067250353 | 6.41E-06 | 0.000287596 |
| TRARG1 | 1.924759312 | 6.56E-06 | 0.000290587 |
| CD24 | 1.031800186 | 6.70E-06 | 0.000293719 |
| C6orf132 | 1.337279459 | 6.85E-06 | 0.00029788 |
| SLC1A4 | -1.082042922 | 6.86E-06 | 0.000297973 |
| C2CD4B | -1.052308514 | 6.96E-06 | 0.000301589 |
| XPNPEP2 | 1.075123104 | 7.30E-06 | 0.000308868 |
| LGALS12 | 1.841952399 | 7.98E-06 | 0.000325875 |
| GALR1 | 1.383766156 | 8.34E-06 | 0.000332562 |
| MGST1 | 1.254813532 | 8.37E-06 | 0.00033268 |
| PCDH10 | 1.18761841 | 8.88E-06 | 0.000341624 |
| DSP | 1.797189964 | 9.35E-06 | 0.000353111 |
| PRKXP1 | 1.303782507 | 9.67E-06 | 0.000361888 |
| ADAD2 | -1.029358889 | 1.04E-05 | 0.000376701 |
| ARHGAP40 | 1.092045652 | 1.06E-05 | 0.000382433 |
| SLC6A10P | -1.070475893 | 1.11E-05 | 0.000392752 |
| SETP21 | 1.066521296 | 1.21E-05 | 0.000412667 |
| ACTG2 | 2.022671501 | 1.30E-05 | 0.000427393 |
| CCL21 | 2.257478243 | 1.32E-05 | 0.000430159 |
| SLCO2A1 | 1.062871226 | 1.58E-05 | 0.000480216 |
| DPP4-DT | 1.016217808 | 1.73E-05 | 0.000507033 |
| LINC00930 | 1.013543947 | 1.77E-05 | 0.000513387 |
| CERS3 | 1.410542119 | 1.79E-05 | 0.000519278 |
| MYEOV | 1.29157996 | 1.85E-05 | 0.000527807 |
| MTCO2P11 | 1.072064725 | 1.89E-05 | 0.000535761 |
| ANGPTL7 | 2.102422656 | 1.95E-05 | 0.000542204 |
| TMEM35A | 1.027050958 | 1.97E-05 | 0.000545645 |
| C1orf127 | -1.023496115 | 2.13E-05 | 0.000570517 |
| MT-TA | -1.300502237 | 2.17E-05 | 0.000574864 |
| CNN1 | 1.271981772 | 2.18E-05 | 0.000575488 |
| FFAR4 | 1.120412143 | 2.50E-05 | 0.000625695 |
| CXCL2 | -1.282128185 | 2.60E-05 | 0.000636233 |
| NUDT19P5 | 1.020325228 | 2.67E-05 | 0.000643271 |
| SNORA35B | 1.080277864 | 2.76E-05 | 0.00065616 |
| XKR9 | -1.264063357 | 2.94E-05 | 0.000680963 |
| MTND1P11 | 1.139486472 | 3.00E-05 | 0.000690748 |
| ZNF365 | 1.031948761 | 3.21E-05 | 0.000721341 |
| GATA6 | 1.038808816 | 3.24E-05 | 0.000726422 |
| PAX5 | -1.097481145 | 3.26E-05 | 0.000728273 |
| LINC01504 | 1.350363222 | 3.40E-05 | 0.000751701 |
| TUBAP4 | 1.053126967 | 3.40E-05 | 0.000751701 |
| LINC01133 | 1.196512448 | 3.68E-05 | 0.000793004 |
| SLC19A3 | 1.732810781 | 3.95E-05 | 0.000830185 |
| TSPAN11 | 1.101126296 | 4.17E-05 | 0.00085624 |
| NSFP1 | 2.17635272 | 4.21E-05 | 0.000860256 |
| AOX3P | 1.036189485 | 4.25E-05 | 0.000863027 |
| DLK1 | -1.052163774 | 4.43E-05 | 0.000883122 |
| DSC3 | 1.891522642 | 4.44E-05 | 0.000884012 |
| MYL12BP3 | -1.010659072 | 4.45E-05 | 0.000884858 |
| ANKRD20A7P | 1.022663954 | 4.45E-05 | 0.000884922 |
| ABCD2 | 1.100108902 | 4.73E-05 | 0.00092177 |
| AZGP1 | 1.723140373 | 4.88E-05 | 0.000936641 |
| U2AF1L5 | -1.891687724 | 4.93E-05 | 0.000940829 |
| APOL5 | -1.28581162 | 4.99E-05 | 0.000945144 |
| TM4SF19 | -1.435646252 | 5.05E-05 | 0.000951494 |
| SCIN | 1.32357329 | 5.29E-05 | 0.000977065 |
| CIB2 | -1.097700763 | 5.32E-05 | 0.000979077 |
| CCBE1 | 1.086980861 | 5.41E-05 | 0.000986718 |
| LINC01982 | -1.061909179 | 5.84E-05 | 0.001035495 |
| OR7A19P | 1.039055222 | 6.14E-05 | 0.001071757 |
| SCD | 2.133508704 | 6.24E-05 | 0.001081942 |
| SLC6A14P3 | 1.443193429 | 6.46E-05 | 0.00110686 |
| ITIH3 | 1.463530977 | 6.80E-05 | 0.001138193 |
| TACR2 | -1.060340294 | 6.94E-05 | 0.00115551 |
| MTCO1P11 | 1.0952685 | 7.25E-05 | 0.001191507 |
| CYP4F24P | 1.25435462 | 7.40E-05 | 0.001207634 |
| U2AF1 | 1.285840605 | 7.66E-05 | 0.001237329 |
| PDE3B | 1.226124768 | 8.04E-05 | 0.00127211 |
| MTRNR2L12 | -1.052493503 | 8.72E-05 | 0.001341243 |
| RPS4XP2 | -1.194120371 | 8.87E-05 | 0.001357422 |
| WSCD2 | 1.010722107 | 9.36E-05 | 0.001406207 |
| EGFEM1P | 1.167783419 | 9.39E-05 | 0.00140837 |
| CILP2 | 1.28460012 | 9.92E-05 | 0.00146661 |
| RPL7P8 | -1.083033987 | 0.00010021 | 0.0014745 |
| LINC02593 | 1.114558223 | 0.000104235 | 0.001519242 |
| TNXA | 1.064399596 | 0.000114031 | 0.00161092 |
| MRPS16P3 | -1.091507719 | 0.000127229 | 0.001738806 |
| TEX41 | 1.190804697 | 0.000132551 | 0.001786364 |
| FAT2 | 1.291432808 | 0.000136784 | 0.001826701 |
| IQCM | -1.02462043 | 0.000136952 | 0.001828318 |
| TWIST2 | 1.091719317 | 0.000151141 | 0.001955505 |
| S100B | 1.421392428 | 0.000155027 | 0.001993213 |
| DDX11 | -1.090739333 | 0.000155175 | 0.001994328 |
| SYT13 | -1.122931762 | 0.000158297 | 0.002016991 |
| RORB | 1.093213384 | 0.000158768 | 0.002018738 |
| SLC7A10 | 1.322704831 | 0.000161072 | 0.002040894 |
| PKHD1L1 | 1.561582049 | 0.000167302 | 0.002098832 |
| ADARB2 | 1.122067317 | 0.000167492 | 0.002099535 |
| CELSR1 | 1.070497235 | 0.000173387 | 0.002153109 |
| LINC01754 | -1.24540304 | 0.000174531 | 0.002161062 |
| GALNT13 | 1.064007453 | 0.000179996 | 0.002205981 |
| TRIM29 | 1.40713648 | 0.000200677 | 0.002380182 |
| TPSD1 | 1.048606595 | 0.000208459 | 0.002442462 |
| ANKRD26P3 | 1.100033898 | 0.000209891 | 0.002451804 |
| POSTN | 1.015850056 | 0.000220031 | 0.002545593 |
| SLITRK2 | 1.000754661 | 0.000261946 | 0.002885215 |
| HEPACAM | 1.288618393 | 0.000265996 | 0.002916512 |
| PDE8B | 1.030024553 | 0.000271224 | 0.002953506 |
| LINC00311 | 1.030682253 | 0.000272578 | 0.002960916 |
| TWIST1 | 1.071996522 | 0.000285257 | 0.003053952 |
| MIR1972-1 | -1.329491057 | 0.000289884 | 0.003089779 |
| LEP | 2.00157599 | 0.000291741 | 0.003097926 |
| LINC00670 | 1.023749099 | 0.000323374 | 0.003329937 |
| ADGRL3 | 1.045566544 | 0.000331018 | 0.003388726 |
| NPIPB2 | 1.041883696 | 0.000333961 | 0.003410753 |
| LINC00960 | 1.068815483 | 0.000373887 | 0.003725834 |
| MARCO | 1.124513981 | 0.000375623 | 0.003738315 |
| ADIPOQ-AS1 | 1.038655971 | 0.000376519 | 0.003745275 |
| POF1B | 1.530413602 | 0.000383847 | 0.003803486 |
| FLG | 2.312505146 | 0.000395982 | 0.003887485 |
| PTPRZ1 | 1.078488964 | 0.00040437 | 0.003943193 |
| RYR2 | 1.005305488 | 0.000449565 | 0.004261045 |
| KRT2 | 2.233826065 | 0.000457698 | 0.004317981 |
| SNX18P7 | 1.209268416 | 0.000465373 | 0.004360546 |
| MYOCD | 1.007127894 | 0.000476933 | 0.004440054 |
| CRTAC1 | 1.032730921 | 0.000485481 | 0.00449153 |
| SNORD115-5 | -1.033972881 | 0.000497072 | 0.004576451 |
| PIK3C2G | 1.073136604 | 0.000534577 | 0.004821688 |
| CCL13 | 1.028695686 | 0.000535752 | 0.004831153 |
| ALKAL2 | 1.060231235 | 0.000589089 | 0.005172718 |
| COPRSP1 | 1.158158165 | 0.000624705 | 0.005403513 |
| NPIPB15 | 1.261162499 | 0.000648839 | 0.005551302 |
| LHCGR | 1.066448163 | 0.000680424 | 0.005752672 |
| OGN | 1.087373935 | 0.000744991 | 0.006159579 |
| MAPK8IP1P2 | -1.68644395 | 0.000746931 | 0.006170335 |
| ITLN1 | 1.578984533 | 0.000751245 | 0.006190575 |
| CALML5 | 1.307480465 | 0.000843267 | 0.006735493 |
| DSG1 | 1.853727134 | 0.000899915 | 0.00705796 |
| EFHB | -1.091427076 | 0.000902275 | 0.007066419 |
| CALB2 | 1.327939513 | 0.000931103 | 0.007217494 |
| TFF3 | 1.076049106 | 0.000939314 | 0.007263638 |
| KRT16P6 | -1.124626046 | 0.001105709 | 0.00823044 |
| MT-TQ | -1.537175199 | 0.001218841 | 0.008875693 |
| SBSPON | 1.116808832 | 0.001227188 | 0.008911256 |
| LY6D | 1.078059397 | 0.001317903 | 0.009394985 |
| KCNA1 | 1.119288979 | 0.001340333 | 0.009518564 |
| LUZP2 | 1.016236587 | 0.001341306 | 0.009521965 |
| TAS2R43 | 1.078868411 | 0.001385734 | 0.009761343 |
| FOS | -1.42298506 | 0.001569398 | 0.0107586 |
| ADH4 | 1.002111283 | 0.001586782 | 0.01083735 |
| RNU2-36P | 1.06268929 | 0.00169918 | 0.011452936 |
| SCEL | 1.310247531 | 0.001907705 | 0.012568776 |
| FOSB | -2.036457566 | 0.001924506 | 0.012653545 |
| DSC1 | 1.537058278 | 0.002021923 | 0.013139608 |
| SCDP1 | 1.421239896 | 0.002062322 | 0.013352678 |
| SBSN | 1.419639895 | 0.002084489 | 0.013469085 |
| DMKN | 1.348624401 | 0.002187113 | 0.014007901 |
| PWRN1 | 1.024091232 | 0.002561729 | 0.015808128 |
| KRT80 | 1.122075757 | 0.002687528 | 0.016427803 |
| TUBB1 | -1.045172934 | 0.003254235 | 0.018980929 |
| BMP7 | 1.016588228 | 0.003284725 | 0.019118375 |
| SLC22A2 | 1.008900062 | 0.003798789 | 0.021403539 |
| MIR503HG | 1.002235343 | 0.004094643 | 0.022666771 |
| SLC5A4 | 1.06755751 | 0.004368218 | 0.023842647 |
| KRT5 | 1.431466747 | 0.004540327 | 0.024594233 |
| RPRML | -1.101050945 | 0.004709509 | 0.025290595 |
| SNORD3B-2 | -1.262779663 | 0.004770007 | 0.025544392 |
| SERPINA12 | 1.044665404 | 0.004878215 | 0.026022763 |
| SERPINA5 | -1.173926311 | 0.004996627 | 0.026500389 |
| SLC44A5 | -1.774143045 | 0.005157405 | 0.027144423 |
| RPL10P6 | -1.330852653 | 0.005405675 | 0.028121316 |
| KRT14 | 1.333105066 | 0.005406578 | 0.028122221 |
| MMRN1 | 1.164013424 | 0.005549338 | 0.028667743 |
| FLG2 | 1.699529953 | 0.005998548 | 0.030506519 |
| PSPHP1 | 2.016426457 | 0.006584582 | 0.032935713 |
| FRMPD2B | 1.158585605 | 0.006928648 | 0.034274693 |
| CXCL8 | 1.142324243 | 0.007661194 | 0.037035498 |
| MT-TE | -1.1157884 | 0.008154932 | 0.038943281 |
| CCDC144A | -1.44445456 | 0.009081304 | 0.042422286 |
| LINC01886 | -1.008803364 | 0.009211728 | 0.042922448 |
| CLDN1 | 1.086098239 | 0.009807053 | 0.045102861 |
| IGFN1 | 1.170424972 | 0.010036175 | 0.04593219 |
| LINC01634 | -1.266557998 | 0.010672959 | 0.048217589 |

**Supplementary File 3**

Immune related genes of decreased skeletal muscle function.

| Gene name | Main function |
| --- | --- |
| CRHR2 | The protein encoded by this gene belongs to the G-protein coupled receptor 2 family, shows high affinity for corticotropin releasing hormone and plays an important role in coordinating the endocrine, autonomic, and behavioral responses to stress and immune challenge. |
| ACKR2 | This gene encodes a beta chemokine receptor which is critical for the recruitment of effector immune cells to the inflammation site. |
| ADIPOQ | This gene encodes an adipokine involved in the control of fat metabolism and insulin sensitivity, with direct anti-diabetic, anti-atherogenic and anti-inflammatory activities. Stimulates AMPK phosphorylation and activation in the liver and the skeletal muscle, enhancing glucose utilization and fatty-acid combustion. |
| FGF18 | The protein encoded by this gene plays an important role in the regulation of cell proliferation, cell differentiation and cell migration. The protein is required for normal ossification and bone development. |
| ANGPTL5 | This gene is a protein coding gene related with functional brain measurement, body height, daytime rest measurement, response to methotrexate and juvenile idiopathic arthritis. |
| ELN | This gene encodes a protein which is a component of elastic fibers. Elastic fibers comprise part of the extracellular matrix and confer elasticity to organs and tissues including the heart, skin, lungs, ligaments, and blood vessels. |
| PLA2G2A | This gene is related with host antimicrobial defense, inflammatory response and tissue regeneration, contributing to lipid remodeling of cellular membranes and generation of lipid mediators. |
| SEMA3D | This gene encodes a member of the semaphorin III family of secreted signaling proteins that are involved in axon guidance during neuronal development. |
| RBP4 | This protein belongs to the lipocalin family and is the specific carrier for retinol (vitamin A alcohol) in the blood. It delivers retinol from the liver stores to the peripheral tissues. |
| PTGER3 | The protein encoded by this gene is a member of the G-protein coupled receptor family and have many biological functions, which involve digestion, nervous system, kidney reabsorption, and uterine contraction activities. |
| PTHLH | The protein encoded by this gene is a critical regulator of cellular and organ growth, development, migration, differentiation and survival and of epithelial calcium ion transport. The protein also regulates endochondral bone development and epithelial-mesenchymal interactions during the formation of the mammary glands and teeth. |
| FGF7P3 | A pseudogene. |
| CCL21 | This gene is related with immunoregulatory and inflammatory processes, inhibits hemopoiesis and stimulates chemotaxis. |
| ANGPTL7 | The protein encoded by this gene enables identical protein binding activity. Involved in negative regulation of vasculature development involved in avascular cornea development in camera-type eye and regulation of extracellular matrix organization. |
| CXCL2 | The protein encoded by this gene produced by activated monocytes and neutrophils and expressed at sites of inflammation. It is a hematoregulatory chemokine, in vitro, suppresses hematopoietic progenitor cell proliferation. |
| AZGP1 | This gene is involved in stimulating lipid degradation in adipocytes and causing the extensive fat losses associated with some advanced cancers. The protein encoded by this gene may bind polyunsaturated fatty acids. |
| S100B | This gene is related with intraocular pressure measurement, viral load, lymphocyte count and leukocyte count. |
| RORB | It is a protein coding gene associated with clock-controlled autophagy in bone metabolism. |
| LEP | This gene encodes a protein that is secreted by white adipocytes into the circulation and plays a major role in the regulation of energy homeostasis, immune and inflammatory responses, hematopoiesis, angiogenesis, reproduction, bone formation and wound healing. |
| MARCO | The protein encoded by this gene is a member of the class A scavenger receptor family and is part of the innate antimicrobial immune system. |
| CCL13 | This gene is related with the attraction of monocytes, lymphocytes, basophils and eosinophils. CCL13 may be involved in the recruitment of monocytes into the arterial wall during the disease process of atherosclerosis, and play a role in the monocyte attraction in tissues chronically exposed to exogenous pathogens. |
| LHCGR | This gene is related with high density lipoprotein cholesterol measurement, polycystic ovary syndrome, body height and body mass index. |
| OGN | The protein encoded by this gene induces ectopic bone formation in conjunction with transforming growth factor beta and may regulate osteoblast differentiation. |
| FOS | The protein encoded by this gene has been implicated as regulators of cell proliferation, differentiation, and transformation. |
| BMP7 | This gene encodes a secreted ligand of the transforming growth factor-beta superfamily of proteins that plays important role in various biological processes, including embryogenesis, hematopoiesis, neurogenesis and skeletal morphogenesis. |
| CXCL8 | The protein encoded by this gene mediates inflammatory response by attracting neutrophils, basophils, and T-cells to clear pathogens and protect the host from infection. |

**Supplementary File 4**

WB source images.

| ADIPOQ β-Actin  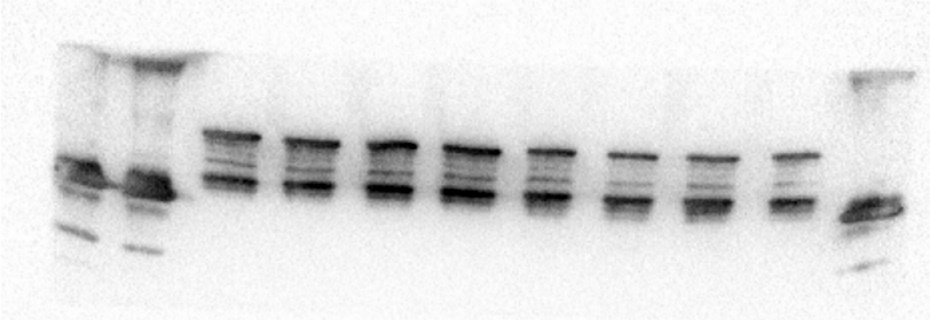  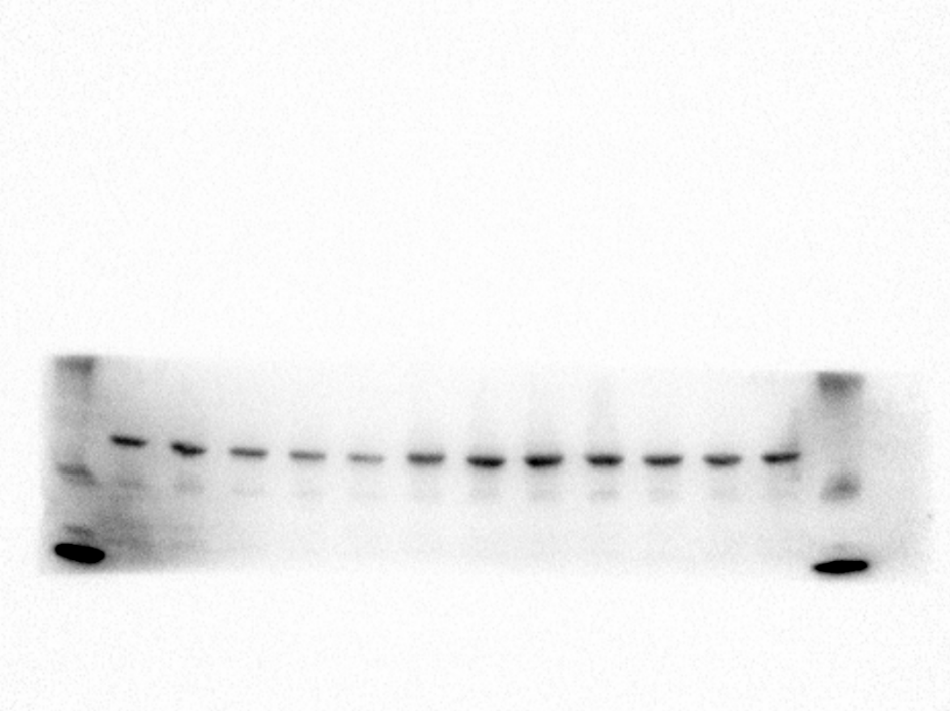 |
| --- |
| ADIPOQ  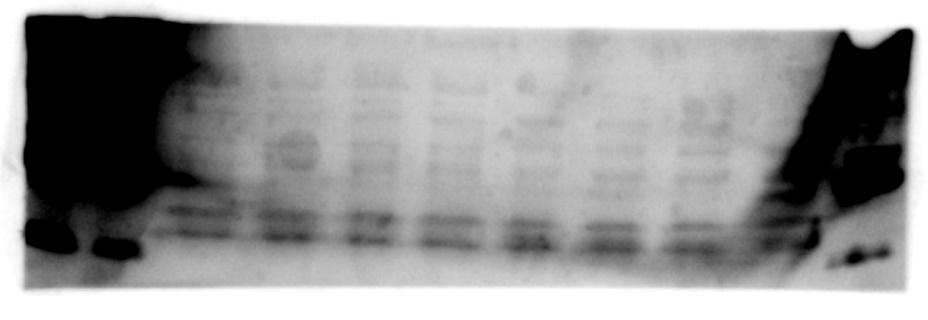  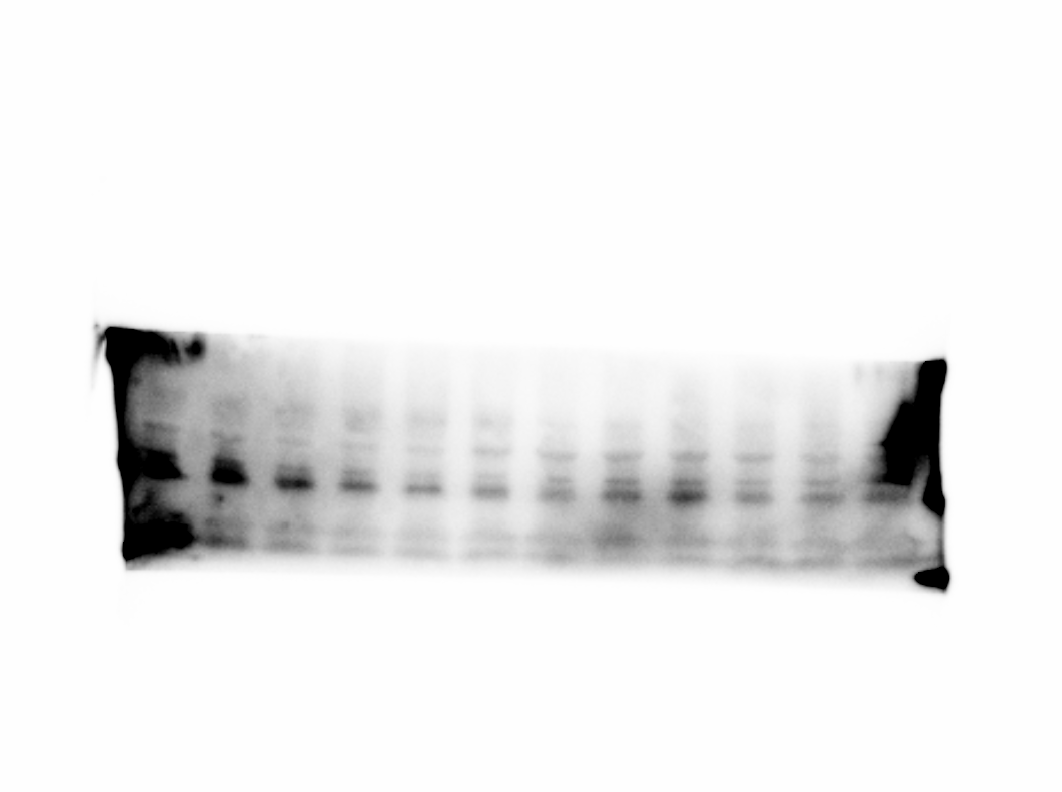 |
| LEP β-Actin  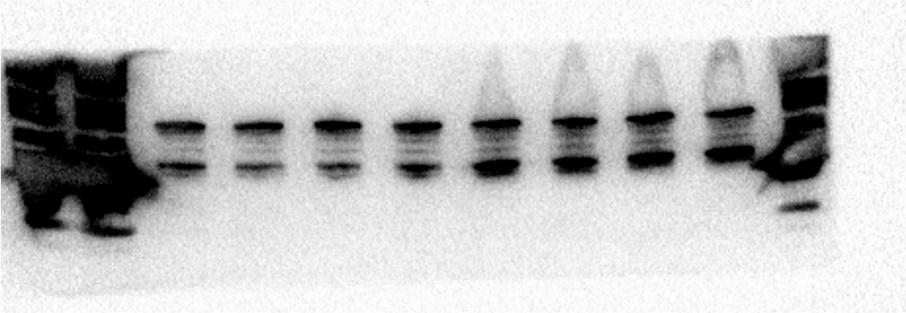  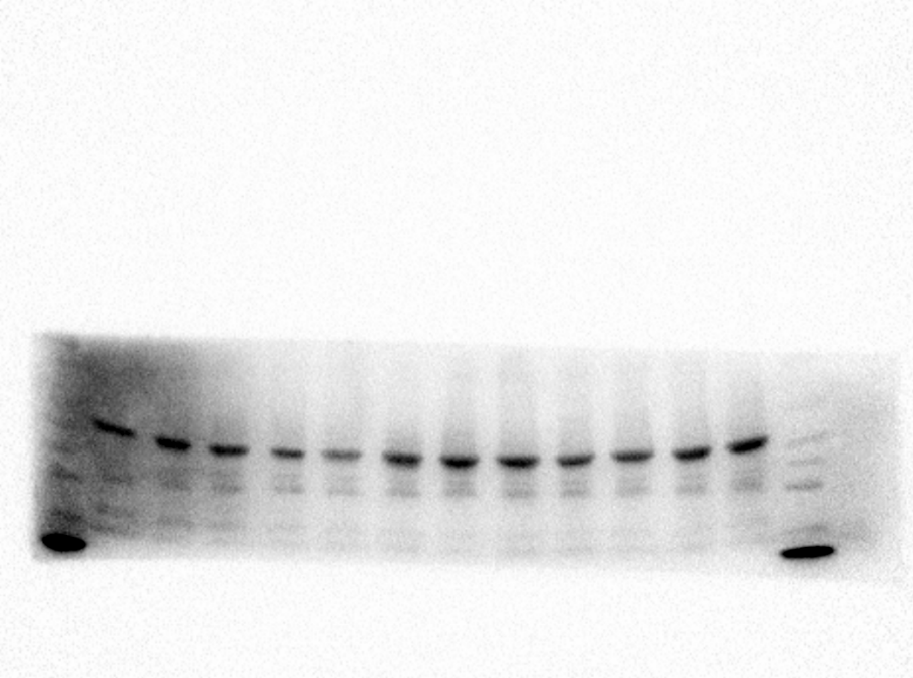 |
| LEP  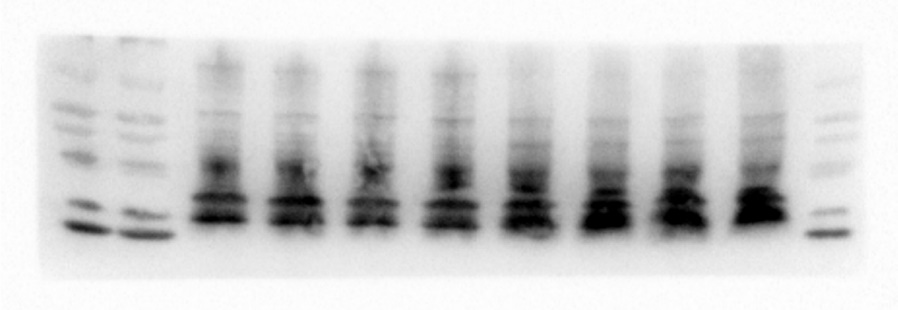  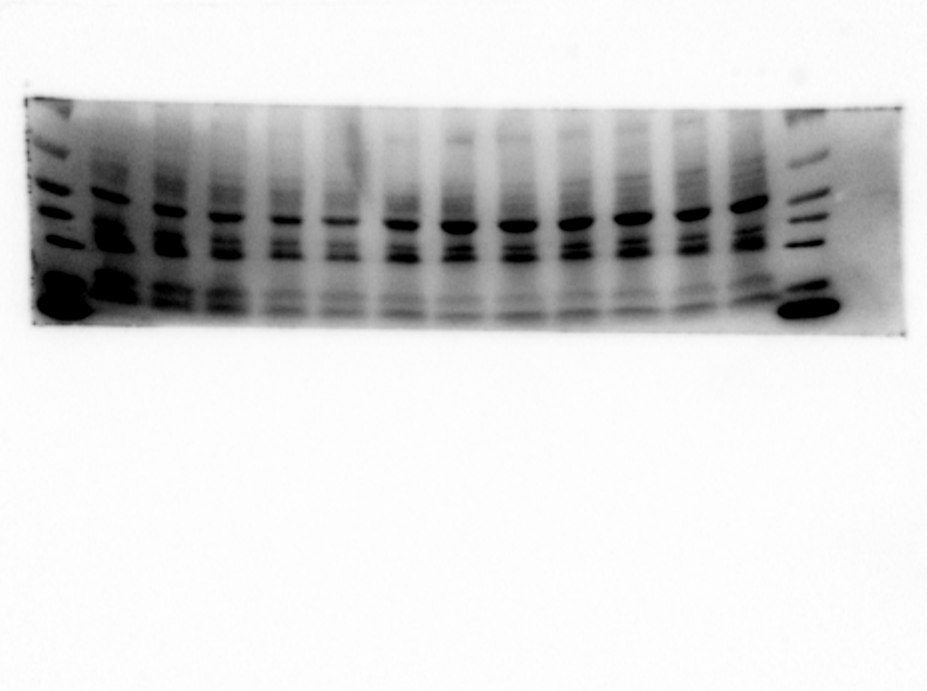 |

Attention: In WB images with 8 strips, left 4 strips belong to Group 1 and right 4 strips belong to Group 2. In WB images with 12 strips, left 6 strips belong to Group 1 and right 6 strips belong to Group 2.
